# Summary from the 2025 International Society for Magnetic Resonance in Medicine workshop on body MRI: Unsolved problems and unmet needs

**DOI:** 10.1002/mrm.70055

**Published:** 2025-09-04

**Authors:** Elizabeth M. Hecht, Houchun Harry Hu, Suraj D. Serai, Holden H. Wu, Ryan L. Brunsing, Alexander R. Guimaraes, Sila Kurugol, Kristina I. Ringe, Ali B. Syed, on behalf of the ISMRM Body MRI Study Group

**Affiliations:** ^1^ Department of Radiology, Weill Cornell Medicine New York New York USA; ^2^ Department of Radiology, Mayo Clinic Jacksonville Florida USA; ^3^ Department of Radiology, Children's Hospital of Philadelphia Philadelphia Pennsylvania USA; ^4^ Department of Radiological Sciences, David Geffen School of Medicine University of California Los Angeles Los Angeles California USA; ^5^ Department of Radiology Stanford University Stanford California USA; ^6^ Department of Diagnostic Radiology, Oregon Health and Sciences University Portland Oregon USA; ^7^ Department of Radiology, Boston Children's Hospital Harvard Medical School Boston Massachusetts USA; ^8^ Department of Diagnostic and Interventional Radiology Hannover Medical School Hannover Germany

**Keywords:** artifacts, artificial intelligence, body MRI, low‐ and high‐field MRI, pediatrics, quantitative imaging, team science

## Abstract

In March of 2025, 145 attendees convened at the Hub for Clinical Collaboration of the Children's Hospital of Philadelphia for the inaugural International Society for Magnetic Resonance in Medicine (ISMRM) Body MRI Study Group workshop entitled “Body MRI: Unsolved Problems and Unmet Needs.” Approximately 24% of the attendees were MD or MD/PhD's, 45% were PhD's, and 30% were early‐career trainees and postdoctoral associates. Among the invited speakers and moderators, 28% were from outside the United States, with a 40:60% female‐to‐male ratio. The 2.5‐day program brought together a multidisciplinary group of scientists, radiologists, technologists, and trainees. Session topics included quantitative imaging biomarkers, low‐ and high‐field strengths, artifact and motion correction, rapid imaging and focused protocols, and artificial intelligence. Another key session focused on the importance of team science and allowed speakers from academia and industry to share their personal experiences and offer advice on how to successfully translate new MRI technology into clinical practice. This article summarizes key points from the event and perceived unmet clinical needs within the field of body MRI.

## 1 INTRODUCTION

The Body MRI Study Group of the International Society for Magnetic Resonance in Medicine (ISMRM) was established in 2023 and aims to facilitate research, promote education, and provides a forum for dialogue between academic and industry partners to foster the development and translation of new MRI technology for abdominal and pelvic imaging that will improve and impact patient care. This inaugural ISMRM‐sponsored Study Group workshop (https://www.ismrm.org/workshops/2025/Body/) was held at the Children's Hospital of Philadelphia's Hub for Clinical Collaboration in Pennsylvania, USA. In addition to invited speakers, 62 proffered abstracts (12 oral, 25 power pitches, and 25 online posters) were accepted. Major themes of the abstracts were liver and kidney function, relaxometry and fat quantification, advanced and robust diffusion‐weighted imaging (DWI), low‐field body MRI, MR fingerprinting (MRF), quantitative imaging biomarkers, artificial intelligence (AI), non‐Cartesian imaging techniques, and the translation of technology into clinical product. Secret audience judges scored and selected the top prizes from the abstract pool (see Table 1).

**TABLE 1:** Oral, power pitch, and poster presentation awardees and travel stipend recipients.^a^

	Institution	Presentation title
Oral presentations		
1st prize Sherya Ramachandran	University of California, Berkeley	Toward Quiet, Free Breathing DCE‐MRI Using Zero TE Imaging
2nd prize Jinjia Chen	New York University	Distortion Free DWI of the Prostate using TGSE‐Based Golden‐Angle PROPELLER Acquisition and Deep Learning Denoising
3rd prize Gastao Cruz	University of Michigan, Ann Arbor	3D breathing T_1_/T_2_/T_2_*/PDFF Kidney Mapping with Dictionary‐Patch Regularized Low‐Rank Motion Corrected Rosetta MRF
Power pitches		
1st prize Jonathan Stelter	Technische Universität München	Simultaneous 3D Free‐Breathing Abdominal Water T_1_ and T_2_ Mapping Using Cartesian Sampling with Spiral Profile Ordering
2nd prize Tom Griesler	University of Michigan, Ann Arbor	Kidney T_1_, T_2_, T_2_*, T1ρ and PDFF Mapping at 0.55 T Using Rosette MRF with Dictionary‐Patch Based Reconstruction
3rd prize Nada Kamona	University of Pennsylvania	Free‐breathing MRI Quantification of Renal Metabolic Rate of Oxygen via Conservation of Flow and Mass
Poster pitches		
1st prize Anika Knupfer	Friedrich‐Alexander‐Universität	Unsupervised Anomaly Detection of Diseases in the Female Pelvis for Real‐Time MR Imaging
2nd prize Corina Margain	University of Texas at Austin	Feasibility of Kidney Blood Volume Quantification Using Non‐Exogenous Contrast Deoxyhemoglobin Dynamic Susceptibility Contrast
3rd prize Elizabeth Huaroc Moquillaza	Technische Universität München	Accelerated Whole Liver Water T_1_ Mapping Using a Neuronal Network‐Based Inversion Recovery Technique
Stipend recipients (in alphabetical order)	Cemre Ariyurek, PhD, Vahid Bazojoo, MD, Jingjia Chen, PhD, Zhongxiu Hu, MSc, Tom Griesler, MSc, Elizabeth Huaroc Moquillaza, MSc, Elima Hussain, PhD, Nada Kamona, MSc, Deniz Kocanaogullari, PhD, Mira Liu, PhD, Haoyang Pei, MSc, Melanie Schellenberg, PhD, Jonathan Stelter, MSc, Jiayi Tang, MSc, Liam Timms, PhD, Aidan Tollefson, MSc, Smiti Tripathy, MSc, Jiachen Wang, BSc, Jiayao Yang, MSc, Ziwei Zhao, PhD, Xuetong Zhou, MSc	

Abbreviations: DCE, dynamic contrast‐enhanced; DWI, diffusion‐weighted imaging; MRF, magnetic resonance fingerprinting; PDFF, proton density fat fraction; TGSE, turbo gradient spin echo.

^a^ Thank you to the judges (Ryan Brunsing, MD, PhD, Hero Hussain, MD, Sila Kurugol, PhD, Thomas Küstner, PhD, Rina Neeman, MD, Jürgen Machann, PhD, Mark Pagel, PhD, James Pipe, PhD, Kristina Ringe, MD, PhD, Amita Shuka‐Dave, PhD, and Holden Wu, PhD) for their time and effort.

Throughout the meeting, attendees were asked to identify and prioritize challenges, opportunities, unsolved problems, and unmet needs in body MRI for future directions of study group activities. This article provides a summary of the event by grouping speaker contributions into several overarching and interconnected themes (see Table 2). Consensus points from roundtable dialogue with vendor panelists are also included. Several clinical review talks are additionally summarized in the attached Supporting Information, along with extensive references provided by each speaker.

**TABLE 2:** List of unsolved problems, unmet needs, and future directions for the ISMRM Body MRI Study Group.

Theme 1: Toward robust, reproducible, and standardized body MRI to promote consistent image quality. Variability and lack of consistency in body MRI protocols and in image quality across facilities still exists and remains a key concern for clinical care and translational research. Research effort and payer reimbursement frameworks for targeted (i.e., problem‐focused, abbreviated) protocols are needed to improve efficiency and patient access. Theme 2: Dedicated focus on pediatric populations and increased accessibility. One size does not fit all. Body MRI solutions that are designed specifically for the pediatric population are needed. Patient access to body MRI remains challenging worldwide, particularly in lower‐income and under‐resourced areas, and especially for pediatric patients. Theme 3: Quantitative imaging biomarkers. Multi‐tissue‐contrast sequences have the potential to improve efficiency for body MRI applications and permit wider adoption of quantitative imaging biomarkers. Repeatable and reproducible quantitative imaging in the body remains challenging, and efforts are needed to harmonize metrics and standards. Recent works in metrology are a step in the right direction. More education is warranted to facilitate clinical translation and vendor involvement is critical. Theme 4: Artificial Intelligence. High‐quality data training is crucial to the success of AI. Real‐world data is superior to simulated data but is logistically difficult to obtain. Optimal AI‐based solutions may, in some cases, require self‐supervised or unsupervised approaches. AI can play a central role in ensuring consistent image quality and reducing variability across patients and facilities, for example, by improving SNR in signal‐poor sequences, facilitating quantitative imaging accuracy and precision, and reducing the dose or removing the need for exogenous contrast agents. AI integration into clinical practice is in its infancy. There is a critical need to develop systematic mechanisms for quality control and quality assurance from the beginning to avoid unintended pitfalls and maximize clinical adoption. Theme 5: Low‐ and high‐field MRI. Low‐field systems can increase patient access and reduce cost. Research effort is needed to validate these systems in comparison to high‐field systems and establish specific use cases. High and ultra‐high field platforms can offer SNR advantages with reasonable safety considerations. Effort is needed to also validate these systems in comparison to status‐quo and establish use cases. AI can assist both low‐ and high‐field systems for body MRI.

Abbreviation: AI, artificial intelligence.

## 2 THEME 1: TOWARD ROBUST, REPRODUCIBLE, AND STANDARDIZED BODY MRI TO PROMOTE CONSISTENT IMAGE QUALITY

Inconsistency of image quality and variability in protocol structure remain central challenges in body MRI. A significant portion of the workshop's discussions focused on quality assurance and quality control to improve consistency in protocols and image quality. In clinical practice, for radiologists and technologists, it is an ongoing challenge to obtain high‐quality images and maintain consistent image quality over time whereas the definition of what constitutes high‐quality imaging remains subjective. The interplay between vendor products, MRI physicists, technologists, physicians, and patients is instrumental to achieving optimal image quality. Lack of medical society guidelines with specific imaging parameters for abdominal (body) MRI means that protocols are still primarily determined by local expertise and personal preferences, which contributes to the wide variability. Although some issues can be resolved through postprocessing methods, the efforts to do so can be time‐consuming and labor‐intensive, and results can be inconsistent. Given the rapid advances in MRI technology, the persistent shortage of expert personnel, and administrative pressure to increase throughput and turnaround time, the ability to obtain consistent high image quality and introduce new innovative pulse sequences remains a difficult balance. There is a need for objective and systematic methods to monitor and address factors that degrade imaging quality proactively and in real time.

Despite major methodological advances in body MRI, Victoria Chernyak, MD, MS (United States), noted that there is still a significant delay in bringing these advances into clinical practice. Factors include the lack of hands‐on experience and dedicated time for staff to learn new techniques. These issues are compounded by the competing demand of increasing clinical volume and the pressure to increase patient throughput. Additionally, many sites use multiple vendors and/or have scanners of varying capabilities depending on the software and systems version, so protocols within and across institutions can be quite variable. Some sequences also remain quite vulnerable to artifacts such as MR cholangiopancreatography and DWI, and results in clinical practice can be inconsistent, even at sites with a high level of MR expertise. She challenged attendees to consider developing a standardized method for reporting of MRI artifacts and other image quality concerns, such that within an institution and across collaborative sites, issues can be tracked and systematically addressed. Additionally, a mechanism to measure the clinical impact of these issues should be developed. Automation of such tools, perhaps with the help of artificial intelligence (AI) to assist technologists, physicists, and radiologists in optimizing image quality and mitigating artifacts, could lead to improved consistency over time.^1^, ^2^, ^3^, ^4^

Leo Razakamanantsoa, MD, PhD (France) discussed how current pelvic MRI protocols are still quite susceptible to motion artifacts, leading to poor image quality, reduced diagnostic confidence, and obscuration of small lesions such as implants from deep pelvic endometriosis and poor delineation of depth of invasion of gynecologic tumors. He echoed the sentiment that a lack of protocol standardization complicates image interpretation and comparison of scans over time. The issue becomes exponentially more difficult when working across multiple institutions on clinical trials and other collaborative efforts. He emphasized that equally important is the standardization of reporting and stressed the need to continue developing reporting guidelines alongside protocol guidelines.^5^, ^6^, ^7^

Kristina Ringe, MD, PhD (Germany) advocated the use of “targeted” or “focused” protocols for applications such as hepatocellular carcinoma and pancreatic cyst surveillance. These are also referred to as “abbreviated” protocols in the literature. Focused protocols are garnering interest as they provide one potential solution to the rising demand for clinical MRI and can help with both increased efficiency of MRI scanner utilization and reduced interpretation burden. She acknowledged that it remains to be determined which sequences should be included in focused protocols and whether exogenous contrast agents will be required. More prospective studies and consensus guidelines are needed to determine the highest yield protocols for specific patient populations and clinical decisions.^8^, ^9^, ^10^

## 3 THEME 2: DEDICATED FOCUS ON PEDIATRIC POPULATIONS AND INCREASED ACCESSIBILITY

Pediatric patients remain an underserved population and require unique imaging solutions. In recent years, the World Federation of Pediatric Imaging has been partnering with the ISMRM through frequent webinars to raise awareness of the importance of dedicated MRI software, hardware, and protocols for young patients. Mary‐Louise Greer, MBBS (Canada), implored attendees to consider addressing patient access, comfort (i.e., noise reduction, free‐breathing), involuntary motion, and image quality (i.e., SNR) when developing MRI technology for the pediatric population. Historically, methodological developments in MRI have originated in adult applications in both neuroradiology and body imaging. This has often led to a gap in access to innovations for pediatric MRI practices. She argued that “one size does not fit all” and challenged the audience to design pediatric‐specific solutions to more effectively address pediatric‐specific needs. She also emphasized that reducing the need for sedation is a key step toward improving access to MRI for pediatric patients worldwide. She shared data from her institution demonstrating how eliminating general anesthesia improves scan efficiency and workflow, lowers cost, and reduces the risk of complications. Several effective methods for reducing the need for anesthesia include scheduling exam times in synchrony with the patient's normal sleep cycle, using feeding and swaddling, MRI simulation, child‐life specialists, animal assistants, and pediatric‐specific headphones and goggles. Additionally, she echoed the theme of using focused and rapid protocols in pediatric patients.^11^, ^12^, ^13^, ^14^, ^15^

Franz Wolfgang Hirsch, MD (Germany), and Farouk Dako, MD (United States), complemented Dr. Greer's presentation. Dr. Hirsch showed real‐time interactive T_2_‐weighted body MRI that exhibited temporal resolutions of 20 ms per image frame. He highlighted examples of imaging the brain, lung, speech, and joint movement in pediatric patients, where both voluntary and involuntary motion are effectively frozen. Additionally, such imaging technology can successfully reduce the use of sedation in pediatric patients.^16^, ^17^, ^18^

Dr. Dako discussed ongoing efforts to address disparities in access to body MRI in Africa through global partnerships. Africa carries 25% of the world's disease burden, and this percentage will likely increase in the next decades. Additionally, Africa has the youngest population globally. According to UNICEF, the continent's pediatric population is expected to reach 1 billion by 2055, such that 40% of the world's children will live in Africa. Currently, the lack of availability of imaging equipment, insufficient infrastructure to operate and maintain equipment and lack of trained personnel limits access to MRI in Africa. He highlighted the educational efforts of the Consortium for Advancement of MRI Education and Research in Africa (CAMERA), the ISMRM African Chapter, and the Medical Image Computing and Computer‐Assisted Intervention Society. These groups have created access to educational materials that outline steps for data management, equipment acquisition, and maintenance. CAMERA is also establishing a global collaborative network for scientists and radiographers, which has led to high‐impact projects and programs to advance radiographer competence and skill sets.^19^, ^20^

## 4 THEME 3: QUANTITATIVE IMAGING BIOMARKERS

Quantitative imaging was an omnipresent theme during the gathering, from DWI‐derived apparent diffusion coefficients (ADC), to T_1_, T_2_, and T_2_* relaxometry, and MRF, to elastography. Matt Hall, PhD (United Kingdom) reviewed the topic of quantitative imaging biomarker (QIB) metrology and discussed ongoing consortia efforts to facilitate the adoption of standards for clinical trials and practice.^21^, ^22^ He emphasized the need to quantify consistency and variability (i.e., uncertainty, bias) as a performance metric for any QIB (i.e., a measurement) using traceable phantoms. By doing so, precision (i.e., repeatability and reproducibility) of QIBs can be rigorously and systematically assessed and documented for appropriate use in clinical trials, especially given the rapid adoption of AI.

Michael Boss, PhD (United States), stressed the importance of having reliable QIBs in multi‐site clinical trials because they can help stratify patient risk, monitor treatment, and assess the efficacy of therapies. For adoption in clinical trials, QIBs must be easy to implement across vendor platforms, and postprocessing analysis must be consistent and standardized for independent core facilities to harmonize and pool results. He reviewed past efforts, currently available resources, and discussed the impact that QIB profiles by The Radiological Society of North America—Quantitative Imaging Biomarkers Alliance (RSNA‐QIBA) have had on the community. He also provided a roadmap for future efforts under the Quantitative Medical Imaging Coalition. He emphasized that current documentary standards can be dense and complex, and in turn, challenging to translate into clinical practice.^23^ To be adopted more readily, these documents need to be practical and amenable to a “cut and paste” approach to facilitate prompt acceptance and incorporation into clinical trials.

Oliver Gurney‐Champion, PhD (The Netherlands), summarized quantitative DWI and DCE MRI with AI using fully connected 1D networks, 2D/3D convolutional neural networks, and recurrent neural networks. Deep learning can offer faster, more accurate, and more robust approaches to QIB estimation compared to conventional methods. Voxelwise prediction of parameters that leverage neighboring spatial information and direct estimation from k‐space raw data were discussed. Echoing other speakers, he indicated that parameter estimation can suffer from poor image quality, which can lead to less meaningful and slower data fitting. Repeatability and reproducibility remain concerning issues in DWI and DCE, and measurable changes that are currently available may be adequate to measure group‐level effects, but are not precise enough for individualized/personalized patient management, which may account for hesitancy in clinical adoption. He further highlighted supervised, surrogate, self‐supervised, and unsupervised learning approaches that aim to improve speed, accuracy, and precision of data fitting and parameter estimation. He cautioned that training only on simulated data may lead to unexpected and erroneous results in real‐world applications. Additionally, underrepresented parameter combinations in training data may lead to inaccuracies, potentially masking pathologies. Current studies focus on single‐center training, raising concerns about generalizability across different scanners and acquisition protocols. Addressing challenges related to variability, generalizability, and domain adaptation will be crucial for widespread clinical adoption.^24^, ^25^, ^26^

Daniel Margolis, MD (United States), complemented Dr. Gurney‐Champion and provided insight into DWI and relaxometry in body applications. Although studies using ADC have shown strong discriminatory value in oncology, challenges remain for widespread adoption, particularly the lack of standardization. He provided examples where ADC has been useful for disease staging and treatment monitoring in patients with prostate, liver, pancreatic, rectal cancer, as well as leiomyosarcoma. On the other hand, there were other conditions where ADC has not shown added value, such as in the assessment of inflammatory bowel disease. He conveyed the need for DWI/ADC phantoms to enable routine quality assurance, especially to account for system drifts and acquisition and reconstruction techniques that are undergoing constant revision.^27^, ^28^, ^29^, ^30^, ^31^

Jürgen Machann, PhD (Germany), summarized developments in AI for quantifying body adipose composition and distribution. AI can significantly reduce the time spent on postprocessing and segmentation. He stressed the importance of fat and iron as QIBs for human health in the context of liver disease and diabetes. Visceral adipose tissue is a strong predictor of metabolic health, with higher amounts suggestive of poor outcomes, such as low insulin sensitivity. Organ fat and iron deposition quantification using chemical‐shift‐encoded water‐fat MRI, T_2_* relaxometry, and quantitative susceptibility mapping methods was reviewed. Relevant examples were given in the liver, heart, and brain. He also discussed the quantification of fatty acid composition, and whilst there has been a steady stream of research works in this area, the degree of triglyceride unsaturation has not yet become a mainstream QIB.^32^, ^33^, ^34^, ^35^

Octavia Bane, PhD (United States), reviewed common inversion‐recovery, Look‐Locker, saturation‐recovery, and variable‐flip‐angle approaches for T_1_ and T_2_ mapping, highlighting advantages and tradeoffs of each technique in liver and kidney applications with a clinical focus on organ function and fibrosis. She emphasized the use of T_1_ mapping in the United Kingdom Biobank study to correlate MRI measurements with genotypes and emphasized the need to establish reference values in control cohorts to account for confounders such as age, gender, etc. She further discussed T_2_ relaxometry in the context of renal inflammation and fibrosis.^36^, ^37^, ^38^

Meng Yin, PhD (United States), updated on the latest advances in MR elastography (MRE), with established applications in liver stiffness and fibrosis, as well as emerging applications in portal hypertension, spleen, stomach, and kidney. Methodological advances in free‐breathing and 3D vector MRE were discussed, facilitating pediatric applications and larger volumetric and whole‐organ coverage. She focused on advanced multiparametric MRE, which enables the assessment of tissue elasticity, viscosity, attenuation, heterogeneity, and anisotropy, widening the application of MRE to fibrosis, inflammation, congestion, and other pathophysiological changes. With multiparametric MRE, she showed examples of how it is now possible to evaluate additional tissue characteristics, such as loss modulus or damping ratio, that measure tissue viscosity or attenuation rate. Integration of these additional mechanical biomarkers is relevant in distinguishing between fibrosis and inflammation, as both conditions may lead to elevated liver stiffness, but have distinct underlying pathophysiological mechanisms.^39^, ^40^

Rasim Boyacioglu, PhD (United States), introduced motion‐compensated MRF for body applications, with a focus on cyclic and non‐rigid motion. He affirmed that in conventional approaches, when motion occurs, only a certain part of k‐space is likely corrupted. However, in the case of MRF, one must consider that the encoded information is corrupting a set of MRF temporal frames with a given signal preparation and tissue contrast. Because motion‐corrupted MRF data deviates from the dictionary, this can lead to biases in downstream parameter estimates. Although motion‐corrupted MRF data can be removed, corrected, re‐acquired, or compensated for during the reconstruction process, different approaches, such as synchronization to body motion, can also be considered. He further introduced MRF acquisitions that are less sensitive to motion, data binning, and with external motion sensors.^41^, ^42^, ^43^, ^44^

Alexander Guimaraes, MD, PhD, and Cory Wyatt, PhD (United States), shared their collaborative work on developing abdominal QIBs in the liver, pancreas, and kidneys using MRF. MRF can overcome some limitations of conventional relaxometry measurements in the abdomen, including respiratory and peristalsis motion, breath‐holding requirements, and long and prohibitive acquisition times. Some of these benefits are particularly useful in assessing the pancreas. Ongoing work includes addressing B_1_ inhomogeneity, radiofrequency (RF) duty cycle, and specific absorption rate limitations in MRF. The team also discussed the use of ferumoxytol to evaluate macrophages and inflammation, including quantitative T_2_ and T_2_* mapping to assess vessel size index and vascular volume fraction.^45^, ^46^

Hansel J. Otero, MD, and Suraj D. Serai, PhD (United States), described their experience with an in‐house developed software at the Children's Hospital of Philadelphia for quantitative body MRI. The software is free and has functionalities for iron and fat quantification, DWI/ADC estimation, tractography, MRE, T_1_, T_2_, and T_2_* relaxometry, DCE perfusion, and volumetric measurements. Examples were shown for liver and kidney applications.^47^, ^48^

## 5 THEME 4: ARTIFICIAL INTELLIGENCE

The existing and potential roles of AI in body MRI were discussed extensively, from data acquisition and reconstruction to motion correction, artifact mitigation, and clinical workflow efficiency. Michael Ohliger, MD, PhD (United States), provided a radiologist's perspective on the emerging use of AI for abdominal MRI and reviewed examples of current applications in clinical practice. He discussed the potential benefits for workflow (e.g., decision support, triage, and second reader), image acquisition and reconstruction, organ segmentation, lesion detection, and tumor characterization. Although AI in body MRI holds immense potential, he cautioned that careful integration is needed to identify and address its limitations while maximizing its benefits for patient care. Several existing limitations were recognized, including lack of transparency in the AI algorithm to the end‐user such that it is difficult to predict failure or false positive results, the reliance on training data that may not encompass a patient cohort under study, the hidden high cost associated with iterative testing and optimizing an AI software for clinical workflow integration, and relatedly, accessibility in low‐resource settings. Additionally, the sensitivity of AI software to inherent variations in MRI scan techniques across vendors and the presence of artifacts need to be explored.^49^, ^50^, ^51^, ^52^

Mariya Doneva, PhD (Germany), provided an overview of the technical considerations needed to successfully bring new AI‐enabled medical devices into clinical practice. There has been a rapid rise in United States Food and Drug Administration‐approved AI‐enabled products in recent years, with a vast majority related to image postprocessing and analysis tools. She identified several key elements required to make AI‐enabled reconstruction algorithms impactful: value proposition, computational efficiency, and generalizable algorithms across diverse populations. Clinical adoption of AI is nascent, with very few devices having accumulated more than 10 000 reimbursement claims. The adoption of AI‐enabled devices is disproportionately concentrated in academic medical centers and high socioeconomic areas. Challenges to adoption include pricing, modified clinical workflows, network connectivity, user trust, and proof of meaningful added value, including improved efficiency, reproducibility, robustness, and benefit to patient outcome.^53^, ^54^

Thomas Küstner, PhD (Germany), outlined several categories of strategies to handle physiological motion and related artifacts. They included suppression of motion, detection and monitoring of motion, triggering and data binning to resolve motion, and avoidance and minimization techniques that exploit data sampling and temporal redundancy in both image‐space and k‐space domains. He asserted that evaluation standards are needed for motion correction and mitigation strategies to enable generalizability, clinical translation, and more widespread adoption.^55^, ^56^, ^57^, ^58^

Efrat Shimron, PhD (Israel), complemented the discussion and described how AI can offer beneficial solutions to body MRI, such as reducing scan duration, improving spatiotemporal resolution, and mitigating motion artifacts. She introduced the concept of implicit “data crimes” in AI from the common use of publicly available databases, which may lead to unknown bias in results. She argued that with judicious application, AI technology will be instrumental in furthering MRI accessibility worldwide, particularly for low‐field applications.^52^, ^59^, ^60^, ^61^

Marcel Dominik Nickel, PhD (Germany) reviewed existing AI‐based physics‐informed image reconstruction and image enhancement techniques. He highlighted several publicly available datasets from academic institutions that can be used for AI algorithm training and reviewed various algorithm design and training strategies, including unrolled end‐to‐end, pre‐trained plug‐and‐play, generative priors, semi‐supervised, self‐supervised, and unsupervised deep neural networks. He further summarized the fundamentals of super‐resolution MRI and recent updates in AI‐enhanced partial Fourier DWI reconstruction. The theme of needing to carefully integrate AI software into the clinical radiology workflow was echoed, along with the judicious choice of training models and datasets to avoid erroneous mimics and hallucinations.^62^, ^63^

Onur Afacan, PhD (United States), discussed susceptibility, motion, noise, flow artifacts, and chemical shift artifacts in body MRI. These artifacts degrade image quality, reduce diagnostic confidence, and hinder quantitative analysis. He noted recent innovations, including dynamic distortion correction via dual‐echo EPI and data binning to correct for motion‐dependent magnetic field inhomogeneity, 3D slice‐to‐volume registration motion correction in EPI‐based DWI, and real‐time motion correction in the abdomen using free induction decay navigators to compensate bulk and non‐rigid motion in DCE MRI. Additionally, he discussed denoising methods and how they may not be effective in the presence of motion. He showed a novel self‐supervised denoising approach using probabilistic models to improve image quality without clean reference data. He conceded that despite recent advances, challenges persist with non‐rigid motion correction approaches, and he emphasized the need for integration of AI with hardware‐based motion tracking to enhance diagnostic accuracy, quantitative reproducibility, and improve cost efficiency.^64^, ^65^, ^66^, ^67^

Arnaud Guidon, PhD (United States), presented a lecture on the pearls and potential pitfalls of applying AI in body MRI. Although AI offers promises of improving workflow efficiency from data acquisition to reconstruction, mitigating motion, increasing the conspicuity of lesions, and reducing noise and artifacts, challenges remain in clinical translation, governance, cost and accessibility, consistency, and reliability. He showed real‐world examples of AI‐enabled denoising, and suppression of Gibbs ringing to enhance boundaries and cautioned users to thoroughly evaluate denoising parameters and their protocol for balancing acquisition speed, spatial resolution, and SNR.^68^, ^69^

Ari Borthakur, PhD (United States), described the development and implementation of a secure cloud‐based AI system for automated CT and MRI image analysis and reporting in radiology at Penn Medicine. The orchestrator software is designed to facilitate the ease of AI software and hardware deployment and use by integrating with existing Picture Archiving and Communication System and medical reporting systems using standardized data formats. The system has been used for body composition analysis and splenomegaly detection in over 15 000 cases to date, and the results are forwarded automatically to radiologists with an average turnaround time of less than 5 min. Results are also integrated into reporting templates and sent to the Penn Medicine biobank repository to enable opportunistic screening and radiomics analysis.^70^, ^71^

## 6 THEME 5: LOW‐ AND HIGH‐FIELD MRI

To complement the growing interest in body MRI across different B_0_ magnetic field strengths and a significant number of abstracts highlighting work at 0.55 T, Clarissa Cooley, PhD (United States), explored the potential of low‐field body MRI. Limited access and the cost of installation, facility, and operation of high‐field MRI units are key reasons why the MR community has been focusing on low‐field systems. Low‐field MRIs are also less limited by the common constraints seen with high‐field scanners, such as susceptibility artifacts (i.e., implants) and safety constraints (i.e., RF heating). Some other advantages of low‐field MRI include lighter weight, smaller footprint, lower power consumption, less acoustic noise generation, better B_1_^+^ homogeneity, shorter T_1_ relaxation times, and slightly longer T_2_ (and T_2_*) relaxation times. She emphasized the critical dependence of SNR on B_0_ field strength. She argues that AI will be especially advantageous for low‐field MRI as it will be able to enhance image quality and improve SNR, and she posits that with appropriate training data, ˜0.05 T body MRI is possible. Although there is significant ongoing research across the ultra‐low‐field (˜0.01 T) to low‐field spectrum (0.1 T), to date, body MRI applications in adults have been successfully demonstrated at 0.55 T in the chest and abdomen and are gaining clinical acceptance and exhibiting diagnostic value. She discussed single‐sided MRI as a possible direction of future development for dedicated low‐field body applications such as prostate, lower spine, and breast, where the magnet is placed on one side of the body, effectively having no bore. Additionally, portable low‐field brain and body MRI may address an unmet need for accessibility and bedside MRI for neonates in the intensive critical unit.^72^, ^73^, ^74^, ^75^, ^76^

Tom Scheenen, PhD (The Netherlands), demonstrated with examples in oncology where staging for primary tumors such as prostate cancer and tracking of lymph nodes metastases can significantly benefit from higher field strengths, because of the higher SNR, finer spatial resolution, and greater sensitivity to iron oxide nanoparticle‐enhanced T_2_*‐weighted imaging. He reminded the audience that at 7 T, there is no RF body coil surrounding the system gantry and that all imaging coils are transmit/receive. RF inhomogeneity, RF power requirements, and patient safety are important points of attention. The community has exploited individual B_1_^+^ shimming and multi‐transmit technology as potential solutions. The strength of MRI of the body at ultra‐high fields will be in magnetization‐prepared pulse sequences with fast low flip angle readouts. He further reviewed developments in free‐breathing radial stack‐of‐stars acquisitions with respiratory navigators and water–fat separation for high spatial resolution 7 T imaging of organs and lymph nodes in the upper abdomen. Prof. Scheenen concluded his talk by reminding the audience that 7 T should be exploited for X‐nuclei imaging, including 31P and 2H for assessing dynamic tumor metabolism.^77^, ^78^, ^79^, ^80^, ^81^

## 7 OUTLOOK: MOVING FORWARD TOGETHER

A highlight of the workshop was a candid roundtable discussion between participants and MRI vendor representatives on how the study group members can more effectively collaborate, engage in team science and industry partnerships, and efficiently translate new imaging technologies into clinical practice. Hersh Chandarana, MD, MBA, and Li Feng, PhD (United States), shared the origin story of the golden‐angle radial sparse parallel (GRASP) technique and discussed their team's decade‐long successful journey of bringing the methodology from benchside into clinical practice for use in a free‐breathing motion‐robust technique for DCE‐MRI. Although GRASP and its variants have been used in over 200 000 cases, the team continues to critically assess and study the limitations of the technique, such that further refinements and optimizations can be made.^82^, ^83^

Richard Ehman, MD (United States), shared inspirational key lessons he learned about advancing MRE from the laboratory bench to standard‐of‐care clinical practice. He conveyed that it is critical to ensure that a new technology or invention is addressing a real‐world problem. Being proactive and engaging with clinical colleagues was emphasized, as was seeking clinical validation and continuously informing stakeholders of developments. He asserted that exclusivity of a technique to a single MRI vendor can limit the widespread dissemination of technology and that it is critical to standardize implementation to enhance generalizability. He further encouraged the audience to investigate partnerships with industry and take advantage of institutional resources that can assist with technology management and transfer. He stressed that collaborations with medical organizations, regulatory agencies, and industry partners can accelerate the advancement of technology into practice and that these bodies should be engaged early in the process.^84^, ^85^, ^86^

## FUNDING INFORMATION

National Institutes of Health, Grant/Award Number: 1R13EB037422‐01.

## Supporting information


**Data S1:** Supporting Information.
