# Supplementary material for: Summary from the 2025 International Society for Magnetic Resonance in Medicine workshop on body MRI: Unsolved problems and unmet needs
Source: Magn Reson Med. 2025 Sep 4;95(1):74–85. doi: 10.1002/mrm.70055 (PMC12620149; doi:10.1002/mrm.70055)
Supplement: Supplementary file 1 — Data S1: Supporting Information. [file MRM-95-74-s001.docx]

**Supplemental Materials**

**ADDITIONAL SUMMARIES of PRESENTATIONS**

**MRI and radiotherapy:**

**Jie Deng, Ph.D.** (United States) discussed the critical role of MRI in radiotherapy treatment of cancer from simulation and treatment planning to assessing and monitoring treatment efficacy. She explained how MRI-guided linear accelerator systems can be used in real-time to formulate treatment plans that can be tailored to adjust for the patient’s evolving response to therapy. Such plans can adapt to the changing size and shape of the target and even manage motion. Magnetic resonance image-guided radiotherapy was demonstrated to provide safe dose escalation treatments, reduce radiation-induced side effects, and improve disease-free survival and long-term patient quality of life. She stressed that a successful radiation oncology service requires coordination and team effort between therapy physicists, oncologists, radiation therapists, MRI physicists, and radiologists. A new American Board of Medical Physics program in Radiation Therapy was introduced.

Prime S, Schiff JP, Hosni A, Stanescu T, Dawson LA, Henke LE. The use of MR-guided radiation therapy for liver cancer. Semin Radiat Oncol. 2024;34:36-44.

Chiloiro G, Gani C, Boldrini L. Rectal cancer MRI guided radiotherapy: a practical review for the physician. Semin Radiat Oncol. 2024;34:64-68.

**Gadolinium contrast agents:**

**Natalie Serkova, Ph.D.** (United States) reviewed the current state-of-the-art in gadolinium-based and non-gadolinium-based contrast agents. She discussed DCE-MRI for tumor staging and treatment response, giving clinical examples in prostate, breast, liver, renal, and brain cancers. She highlighted existing challenges in heterogeneous protocols and kinetics modeling, non-standardized clinical cut-off action points, and how DCE time curves are often still evaluated qualitatively by radiologists, even though permeability and perfusion are important QIBs for cytostatic anticancer agents. Dr. Serkova discussed the use of iron oxide nanoparticles in lymph node imaging, vessel size imaging, macrophage imaging.

Fennessy FM, Fedorov A, Vangel MG, et al. Multiparametric MRI as a biomarker of response to neoadjuvant therapy for localized prostate cancer: a pilot study. Acad Radiol. 2020;27:1432-1439.

Tavakoli AA, Hielscher T, Badura P, et al. Contribution of dynamic contrast-enhanced and diffusion MRI to PI-RADS for detecting clinically significant prostate cancer. Radiology. 2023;306:186-199.

**Beyond gadolinium contrast agents:**

**Mark Pagel, Ph.D.** (United States) demonstrated animal studies imaging extracellular pH of the tumor microenvironment using AcidoCEST, showing that changes in pH can be sensitive to early tumor responses to chemo-, radio, and immunotherapies. He explained an approach to measuring tumor pH using PET/MRI, where an 18F and Gd co-agent dependent on pH was developed such that the change in T1 relaxation of tissue that is caused by the agent reflects the pH and concentration of the agent. He also discussed dynamic MRF for T1 relaxometry that was developed for small animal imaging. Lastly, Dr. Pagel highlighted the challenge of DCE-MRI and its reliance on using separate arterial input functions and argued that using a muscle reference region or MRF can be an alternative.

Lindeman LR, Jones KM, High RA, Howison CM, Shubitz LF, Pagel MD. Differentiating lung cancer and infection based on measurements of extracellular pH with acid CEST MRI. Sci Rep. 2019;9:13002.

Pollard AC, de la Cerda J, Schuler FW, et al. Radiometal-Based PET/MRI Contrast Agents for Sensing Tumor Extracellular pH. Biosensors (Basel). 2022;12:134.

**Whole body MRI in oncology**

**Vipul Sheth, M.D., Ph.D.** (United States) demonstrated advantages of whole-body simultaneous PET/MRI in oncology. The combination of soft tissue contrast, reduced radiation dose, and metabolic and functional capabilities makes PET/MRI attractive. Examples of using hepatobiliary contrast agents in combination with Ga-68 DOTATATE in patients with neuroendocrine tumors were shown. Additionally, high-resolution prostate MRI with prostate-specific membrane antigen PET has revolutionized prostate cancer care and management. Challenges with access to PET/MRI technology, insurance reimbursement, shorter scan times, consistent image quality, and staff expertise remain.

Renzulli M, Clemente A, Ierardi AM, et al. Imaging of Colorectal Liver Metastases: New developments and pending issues. Cancers (Basel). 2020;12:151.

Kulkarni NM, Soloff EV, Tolat PP, et al. White paper on pancreatic ductal adenocarcinoma from the society of abdominal radiology’s disease-focused panel for pancreatic ductal adenocarcinoma: Part I, AJCC staging system, NCCN guidelines, and borderline resectable disease. Abdom Radiol. 2020;45:716–728.​

Kulkarni NM, Mannelli L, Zins M, et al. White paper on pancreatic ductal adenocarcinoma from society of abdominal radiology’s disease-focused panel for pancreatic ductal adenocarcinoma: Part II, update on imaging techniques and screening of pancreatic cancer in high-risk individuals. Abdom Radiol. 2020;45:729–742.​

**Whole body MRI screening in low-risk populations**

**Stella Kang, M.D.** (United States) explored the topic of whole-body screening MRI in the low health risk population and focused on three tenets (a) the implications of incidental findings in this patient population, (b) meeting the needs of patients with a wide variety of health-related preferences and concerns, and (c) the cascade of costs and potential harms to participants and the healthcare system.

Irving G, Holden J. The time-efficiency principle: time as the key diagnostic strategy in primary care. Fam Pract. 2013;30:386-379.

Scherer LD, Caverly TJ, Burke J, et al. Development of the medical maximizer-minimizer scale. Health Psychol. 2016;35:1276-1287

Kang SK, Gulati R, Moise N, Hur C, Elkin E. Multi-cancer early detection tests: state of the art and implications for radiologists. Radiology; 2025;314:e233448.

Kang SK, Scherer LD, Megibow AJ, et al. A randomized study of patient risk perception for incidental renal findings on diagnostic imaging tests. AJR Am J Roentgenol. 2018;210:369-375​.

**Current hot topics in clinical body MRI**

**Carla Harmath, M.D.** (United States) discussed recent advancements in liver-directed therapies, including ablative therapies and radiation-based techniques. She also summarized systemic therapies such as tyrosine kinase inhibitors and immune checkpoint inhibitors. She discussed standardized methods of assessment and reporting, including LI-RADS treatment algorithms. Several unmet needs were identified, including addressing variability in the interpretation of post-treatment changes due to the presence of post-procedural perfusion or inflammatory changes, and encouraging the development of MRI methods and biomarkers that could more effectively predict response to therapy and more systematically guide clinical management decisions.

Mendiratta-Lala M, Aslam A, Maturen KE, et al. LI-RADS treatment response algorithm: performance and diagnostic accuracy with radiologic-pathologic explant correlation in patients with SBRT-treated hepatocellular carcinoma. Int J Radiat Oncol Biol Phys. 2022;112:704-714.

Hamad A, Aziz H, Kamel IR, Diaz DA, Pawlik TM. Yttrium-90 radioembolization: current indications and outcomes. J Gastrointest Surg Off J Soc Surg Aliment Tract. 2023;27:604-614.

Dadrass F, Sher A, Kim E. Update on locoregional therapies for liver cancer: radiation segmentectomy. Curr Oncol. 2023;30:10075-10084.

Gutman MJ, Serra LM, Koshy M, Katipally RR. SBRT for Liver Tumors: what the interventional radiologist needs to know. Semin Interv Radiol. 2024;41:1-10.

Sarwar A, Malik MS, Vo NH, et al. Efficacy and safety of radiation segmentectomy with 90Y resin microspheres for hepatocellular carcinoma. Radiology. 2024;311:e231386.

**Maria El Homsi, M.D.** (United States) showed case examples of how MRI plays a critical role in the local staging, restaging, surveillance, and risk stratification of patients with rectal cancer. In recent years, more treatment options are available and select patients are managed under a “watch and wait” approach that eliminates the need for surgery and preserves rectal function. These patients are closely monitored using endoscopy and MRI. Total neoadjuvant therapy remains the standard treatment approach for stage II and III rectal cancer, where chemotherapy and radiation are given before surgery. MRI assists treatment planning decisions and ensures the most appropriate use of neoadjuvant therapy to achieve optimal patient outcome. Dr. El Homsi echoed the sentiments that radiologists need to implement standardized imaging protocols and use structured reporting in rectal cancer management to communicate more effectively with surgeons and oncologists.

Modena Heming CA, Alvarez JA, Miranda J, Cardoso D, Almeida Ghezzi CL, Nogueira GF, Costa-Silva L, Damasceno RS, Morita TO, Smith JJ, Horvat N. Mastering rectal cancer MRI: From foundational concepts to optimal staging. Eur J Radiol. 2025 Feb;183:111937.

El Homsi M, Bercz A, Chahwan S, et al. Watch & wait - Post neoadjuvant imaging for rectal cancer. Clin Imaging. 2024;10:110166.

**Valdair Francisco Muglia, M.D.** (Brazil) discussed how the widespread adoption of PI-RADS (Prostate Imaging Reporting and Data System) has elevated the role of MRI in the management of prostate cancer. PI-RADS has promoted standardization of image acquisition, interpretation, and risk stratification. With the expansion of clinical indications for MRI, new scoring systems and algorithms continue to be developed and validated. He discussed the prostate cancer radiological estimation of change in sequential evaluation (PRECISE) recommendations for assessment of disease progression in patients on active surveillance, PI-RR (Prostate Imaging for Recurrence Reporting) for recurrence post-radiation, PI-FAB (Prostate Imaging after Focal Ablation) and the Trans Transatlantic Recommendations for Prostate Gland Evaluation with MRI after Focal Therapy (TARGET) for assessment of response to focal ablative therapy and the image quality scoring system (PI-QUAL).

Turkbey B, Rosenkrantz AB, Haider MA, et al. Prostate imaging reporting and data system version 2.1: 2019 update of prostate imaging reporting and data system version 2. Eur Urol. 2019;76:340-351.

Panebianco V, Villeirs G, Weinreb JC, et al. Prostate magnetic resonance imaging for local recurrence reporting (PI-RR): International consensus-based guidelines on multiparametric magnetic resonance imaging for prostate cancer recurrence after radiation therapy and radical prostatectomy. Eur Urol Oncol. 2021;4:868-876.

Park KJ, Choi SH, Kim MH, Kim JK, Jeong IG. Performance of prostate imaging reporting and data system version 2.1 for diagnosis of prostate cancer: a systematic review and meta-analysis. J Magn Reson Imaging. 2021;54:103-112.

**Kimberly Shampain, M.D.** (United States) elaborated on MRI of acute adnexa. She reviewed commonly used MRI protocols for the female pelvis, with a particular focus on utilizing b-values of 1000 and 1400 s/mm^2^ in DWI to suppress the bladder to help identify soft tissue components within adnexal masses, lymph nodes, and peritoneal deposits. She illustrated MRI cases of acute pelvic inflammatory disease, ovarian and fallopian tube torsion, and ovarian hyperstimulation, emphasizing that prompt diagnosis in acute adnexa is important to prevent serious complications such as more widespread infection or ovarian infarction.

Rockall AG, Jalaguier-Coudray A, Thomassin-Naggara I. MR imaging of the adnexa: technique and imaging acquisition. Magn Reson Imaging Clin N Am. 2023;31:149-161.

Stein EB, Shampain KL. Magnetic resonance imaging of acute adnexal pathology. Magn Reson Imaging Clin N Am. 2023;31:109-120.

**Supplemental Materials**

**ADDITIONAL REFERENCES BY SPEAKERS (in alphabetical order of last name)**

***Afacan***

Schindera ST, Merkle EM, Dale BM, Delong DM, Nelson RC. Abdominal magnetic resonance imaging at 3.0 T: what is the ultimate gain in signal-to-noise ratio? Acad Radiol. 2006;13:1236-1243. PMID: 16979073

Brau AC, Brittain JH. Generalized self-navigated motion detection technique: preliminary investigation in abdominal imaging. Magn Reson Med. 2006;55:263-270. PMID: 16408272

Kober T, Marques JP, Gruetter R, Krueger G. Head motion detection using FID navigators. Magn Reson Med. 2011;66:35-143. PMID: 21337424

Manjón JV, Coupé P, Concha L, Buades A, Collins DL, Robles M. Diffusion weighted image denoising using overcomplete local PCA. PLoS One. 2013;8:e73021. PMID: 24019889

Zaitsev M, Maclaren J, Herbst M. Motion artifacts in MRI: A complex problem with many partial solutions. J Magn Reson Imaging. 2015;42:887-901. PMID: 25630632

Veraart J, Novikov DS, Christiaens D, Ades-Aron B, Sijbers J, Fieremans E. Denoising of diffusion MRI using random matrix theory. Neuroimage. 2016;142:394-406. PMID: 27523449

Afacan O, Hoge WS, Wallace TE, Gholipour A, Kurugol S, Warfield SK. Simultaneous motion and distortion correction using dual-echo diffusion-weighted MRI. J Neuroimaging. 2020;30:276-285. PMID: 32374453

Coll-Font J, Afacan O, Chow JS, et al. Bulk motion‐compensated DCE‐MRI for functional imaging of kidneys in newborns. J Magn Reson Imaging. 2020;52:207-216. PMID: 31837071

Zöllner FG, Šerifović-Trbalić A, Kabelitz G, Kociński M, Materka A, Rogelj P. Image registration in dynamic renal MRI-current status and prospects. MAGMA. 2020;33(1):33-48. PMID: 31598799

Ariyurek C, Wallace TE, Kober T, Kurugol S, Afacan O. Prospective motion correction in kidney MRI using FID navigators. Magn Reson Med. 2023;89:276-285. PMID: 36063497

Gilani N, Mikheev A, Brinkmann IM, et al. Characterization of motion dependent magnetic field inhomogeneity for DWI in the kidneys. Magn Reson Imaging. 2023;100:93-101. PMID: 36924807

Vasylechko S, Afacan O, Kurugol S. Self supervised denoising diffusion probabilistic models for abdominal DW-MRI. Comput Diffus MRI. 2023;14328:80-91. PMID: 38736559

***Bane***

Piechnik SK, Ferreira VM, Dall'Armellina E, et al. Shortened Modified Look-Locker Inversion recovery (ShMOLLI) for clinical myocardial T1-mapping at 1.5 and 3 T within a 9 heartbeat breathhold. Journal of Cardiovascular Magnetic Resonance. 2010;12:69. PMID: 21092095

Roujol S, Weingärtner S, Foppa M, et al. Accuracy, precision, and reproducibility of four T1 mapping sequences: a head-to-head comparison of MOLLI, ShMOLLI, SASHA, and SAPPHIRE. Radiology. 2014;272:683-689. PMID: 24702727

Banerjee R, Pavlides M, Tunnicliffe EM, et al. Multiparametric magnetic resonance for the non-invasive diagnosis of liver disease. J Hepatol. 2014;60:69-77. PMID: 24036007

Hoad CL, Palaniyappan N, et al. A study of T1 relaxation time as a measure of liver fibrosis and the influence of confounding histological factors. NMR Biomed. 2015;28:706-714. PMID: 25908098

Yoon JH, Lee JM, Kim E, Okuaki T, Han JK. Quantitative liver function analysis: volumetric T1 mapping with fast multi section B1 inhomogeneity correction in hepatocyte-specific contrast-enhanced liver MR imaging. Radiology. 2016;282:408-417. PMID: 27697007

Bane O, Hectors SJ, Wagner M, et al. Accuracy, repeatability, and interplatform reproducibility of T(1) quantification methods used for DCE-MRI: Results from a multicenter phantom study. Magn Reson Med. 2018;79:2564-2575. PMID: 28913930

Kim JW, Lee YS, Park YS, et al. Multiparametric MR index for the diagnosis of non-alcoholic steatohepatitis in patients with non-alcoholic fatty liver disease. Sci Rep. 2020;10:2671. PMID: 32060386

Harrison SA, Rossi SJ, Paredes AH, et al. NGM282 improves liver fibrosis and histology in 12 weeks in patients with nonalcoholic steatohepatitis. Hepatology. 2020;71:1198-1212. PMID: 30805949

Schaapman JJ, Tushuizen ME, Coenraad MJ, Lamb HJ. Multiparametric MRI in patients with nonalcoholic fatty liver disease. J Magn Reson Imaging. 2021;53:1623-1631. PMID: 32822095

Altinmakas E, Bane O, Hectors SJ, et al. Performance of native and gadoxetate-enhanced liver and spleen T(1) mapping for noninvasive diagnosis of clinically significant portal hypertension: preliminary results. Abdom Radiol (NY). 2022;47:3758-3769. PMID: 36085378

von Ulmenstein S, Bogdanovic S, Honcharova-Biletska H, et al. Assessment of hepatic fibrosis and inflammation with looklocker T1 mapping and magnetic resonance elastography with histopathology as reference standard. Abdom Radiol (NY). 2022;47:3746-3757. PMID: 36038643

Bane O, Hector SJ, Gordic S, et al. Multiparametric magnetic resonance imaging shows promising results to assess renal transplant dysfunction with fibrosis. Kidney Int. 2020;97:414-420. PMID: 31874802

***Borthakur***

Chae A, Yao MS, Sagreiya H, et al. Strategies for implementing machine learning algorithms in the clinical practice of radiology. Radiology. 2024;310:e223170. PMID: 38259208

Kim J, Chae A, Duda J, et al. Automated Characterization of Abdominal MRI Exams Using Deep Learning. Preprint. *Res Sq*. 2024;rs.3.rs-5334453. Published 2024 Dec 9. doi:10.21203/rs.3.rs-5334453/v1 PMID: 39711527

Song I, Thompson EW, Verma A, et al. Clinical correlates of CT imaging-derived phenotypes among lean and overweight patients with hepatic steatosis. Sci Rep. 2024;14:53. PMID: 38167550

MacLean MT, Jehangir Q, Vujkovic M, et al. Quantification of abdominal fat from computed tomography using deep learning and its association with electronic health records in an academic biobank. J Am Med Inform Assoc. 2021;28:1178-1187. PMID: 33576413

***Boyacioglu***

Jiang Y, Ma D, Seiberlich N, Gulani V, Griswold MA. MR fingerprinting using fast imaging with steady state precession (FISP) with spiral readout. Magn Reson Med. 2015;74:1621-1631. PMID: 25491018

Yu AC, Badve C, Ponsky LE, et al. Development of a combined MR fingerprinting and diffusion examination for prostate cancer. Radiology. 2017;283;729–738. PMID: 28187264

Yu Z, Zhao T, Assländer J, Lattanzi R, Sodickson DK, Cloos MA. Exploring the sensitivity of magnetic resonance fingerprinting to motion. Magn Reson Imaging. 2018;54:241–248. PMID: 30193953

Mehta BB, Ma D, Pierre EY, Jiang Y, Coppo S, Griswold MA. Image reconstruction algorithm for motion insensitive MR fingerprinting: MORF. Magn Reson Med. 2018;80:2485–2500. PMID: 29732610

Cruz G, Jaubert O, Schneider T, Botnar RM, Prieto C. Rigid motion-corrected magnetic resonance fingerprinting. Magn Reson Med. 2019;81:947–961. PMID: 30229558

Poorman ME, Martin Mn, Ma D, et al. Magnetic resonance fingerprinting Part 1: Potential uses, current challenges, and recommendations. J Magn Reson Imaging. 2020;51:675-692. PMID: 31264748

van Riel MHC, Yu Z, Hodono S, Xia D, Chandarana H, Fujimoto K, Cloos MA. Free-breathing abdominal T1 mapping using an optimized MR fingerprinting sequence. NMR Biomed. 2021;34:e4531. PMID: 33902155

Huang SS, Boyacioglu R, Bolding R, MacAskill C, Chen Y, Griswold MA. Free-breathing abdominal magnetic resonance fingerprinting using a pilot tone navigator. J Magn Reson Imaging. 2021;54:1138-1151. PMID: 33949741

Vahle T, Bacher M, Rigie D, et al. Respiratory motion detection and correction for MR using the pilot tone: application for MR and simultaneous PET/MR examinations. Invest Radiol. 2020;55:153-159. PMID: 31895221

Solomon E, Rigie DS, Vahle T, et al. Free-breathing radial imaging using a pilot-tone radiofrequency transmitter for detection of respiratory motion. Magn Reson Med. 2021;85:2672–2685. PMID: 33306216

Huang SS, Boyacioglu R, Bolding R, MacAskill C, Chen Y, Griswold MA. Free-breathing abdominal magnetic resonance fingerprinting using a pilot tone navigator. J Magn Reson Imaging. 2021; 54:1138–1151. PMID: 33949741

Wang N, Cao T, Han F, et al. Free-breathing multitasking multi-echo MRI for whole-liver water-specific T1, proton density fat fraction, and quantification. Magn Reson Med. 2022;87:120-137. PMID: 34418152

***Chandarana and Feng***

Xue Y, Yu J, Kang HS, Englander S, Rosen MA, Song HK. Automatic coil selection for streak artifact reduction in radial MRI. Magn Reson Med. 2012;67:470-6. PMID: 21656562

Cheng JY, Zhang T, Ruangwattanapaisarn N, Alley MT, Uecker M, Pauly JM, Lustig M, Vasanawala SS. Free-breathing pediatric MRI with nonrigid motion correction and acceleration. J Magn Reson Imaging. 2015;42:407-20. PMID: 25329325

Chen Y, Lee GR, Wright KL, Badve C, Nakamoto D, Yu A, Schluchter MD, Griswold MA, Seiberlich N, Gulani V. Free-breathing liver perfusion imaging using 3-dimensional through-time spiral generalized autocalibrating partially parallel acquisition acceleration. Invest Radiol. 2015;50:367-375. PMID: 25946703

Feng L, Axel L, Chandarana H, Block KT, Sodickson DK, Otazo R. XD-GRASP: Golden-angle radial MRI with reconstruction of extra motion-state dimensions using compressed sensing. Magn Reson Med. 2016;75:775-788. PMID: 25809847

Feng L, Benkert T, Block KT, Sodickson DK, Otazo R, Chandarana H. Compressed sensing for body MRI. J Magn Reson Imaging. 2017;45:966-987. PMID: 27981664

Feng L, Huang C, Shanbhogue K, Sodickson DK, Chandarana H, Otazo R. RACER-GRASP: Respiratory-weighted, aortic contrast enhancement-guided and coil-unstreaking golden-angle radial sparse MRI. Magn Reson Med. 2018;80:77-89. PMID: 29193260

Armstrong T, Ly KV, Murthy S, Ghahremani S, Kim GHJ, Calkins KL, Wu HH. Free-breathing quantification of hepatic fat in healthy children and children with nonalcoholic fatty liver disease using a multi-echo 3-D stack-of-radial MRI technique. Pediatr Radiol. 2018;48:941-953. PMID: 29728744

Mandava S, Keerthivasan MB, Martin DR, Altbach MI, Bilgin A. Radial streak artifact reduction using phased array beamforming. Magn Reson Med. 2019;81:3915-3923. PMID: 30756432

Ong F, Zhu X, Cheng JY, et al. Extreme MRI: Large-scale volumetric dynamic imaging from continuous non-gated acquisitions. Magn Reson Med. 2020;84:1763-1780. PMID: 32270547

Serai SD, Hu HH, Ahmad R, White S, Pednekar A, Anupindi SA, Lee EY. Newly developed methods for reducing motion artifacts in pediatric abdominal MRI: Tips and Pearls. AJR Am J Roentgenol. 2020;214:1042-1053. PMID: 32023117

Feng L, Wen Q, Huang C, Tong A, Liu F, Chandarana H. GRASP-Pro: imProving GRASP DCE-MRI through self-calibrating subspace-modeling and contrast phase automation. Magn Reson Med. 2020;83:94-108. PMID: 31400028

Coll-Font J, Afacan O, Chow JS, Lee RS, Stemmer A, Warfield SK, Kurugol S. Bulk motion-compensated DCE-MRI for functional imaging of kidneys in newborns. J Magn Reson Imaging. 2020;52:207-216. PMID: 31837071

Fu Z, Johnson K, Altbach MI, Bilgin A. Cancellation of streak artifacts in radial abdominal imaging using interference null space projection. Magn Reson Med. 2022;88:1355-1369. PMID: 35608238

Feng L. 4D Golden-Angle Radial MRI at Subsecond Temporal Resolution. NMR Biomed. 2023 ;36:e4844. PMID: 36259951

Feng L. Live-view 4D GRASP MRI: A framework for robust real-time respiratory motion tracking with a sub-second imaging latency. Magn Reson Med. 2023;90:1053-1068. PMID: 37203314

***Cherniak***

Koh DM, Collins DJ. Diffusion-weighted MRI in the body: applications and challenges in oncology. AJR Am J Roentgenol. 2007;188:1622-1635. PMID: 17515386

Koh DM, Blackledge M, Collins DJ, et al. Reproducibility and changes in the apparent diffusion coefficients of solid tumours treated with combretastatin A4 phosphate and bevacizumab in a two-centre phase I clinical trial. Eur Radiol. 2009;19:2728-2738. PMID: 19547986

Kakite S, Dyvorne H, Besa C, et al. Hepatocellular carcinoma: short-term reproducibility of apparent diffusion coefficient and intravoxel incoherent motion parameters at 3.0T. J Magn Reson Imaging. 2015;41:149-156. PMID: 24415565

Kierans AS, Rosenkrantz AB. Radial T1-weighted magnetic resonance imaging: Background, clinical applications, and future directions. Appl Radiol. 2016;5:24-33. link

Taouli B, Beer AJ, Chenevert T, et al. Diffusion-weighted imaging outside the brain: Consensus statement from an ISMRM-sponsored workshop. J Magn Reson Imaging. 2016;44:521-540. PMID: 26892827

***Cooley***

Zhao Y, Ding Y, Lau V, et al.. Whole-body magnetic resonance imaging at 0.05 Tesla. Science. 2024; 384(6696):eadm7168. doi: 10.1126/science.adm7168. PMID: 38723062

Campbell-Washburn AE, Malayeri AA, Jones EC, et al. T2-weighted lung imaging using a 0.55-T MRI system. Radiol Cardiothorac Imaging. 2021;3:e200611. PMID: 34250492

Ramachandran A, Hussain HK, Gulani V, et al. Abdominal MRI on a commercial 0.55T system: initial evaluation and comparison to higher field strengths. Acad Radiol. 2024;31:3177-3190. PMID: 38320946

Bandettini WP, Shanbhag SM, Mancini C, et al. A comparison of cine CMR imaging at 0.55 T and 1.5 T. J Cardiovasc Magn Reson. 2020;22:37. PMID: 32423456

Selvaganesan K, Wan Y, Ha Y, et al. Magnetic resonance imaging using a nonuniform Bo (NuBo) field cycling magnet. PLoS One. 2023;18(6):e0287344. PMID: 37319289

***Dako***

Ezzati M, Pearson-Stuttard J, Bennett JE, Mathers CD. Acting on non-communicable diseases in low- and middle-income tropical countries. Nature. 2018;559:507-516. PMID: 30046068

Warnert EAH, Nayak K, Menon R, et al. Resonate: Reflections and recommendations on implicit biases within the ISMRM. J Magn Reson Imaging. 2019;49:1509-1511. PMID: 30666751

Warnert EAH, Kasper L, Meltzer CC, et al. Resonate: Reaching excellence through equity, diversity, and inclusion in ISMRM. J Magn Reson Imaging. 2021;53:1608-1611. PMID: 33350020

Kazerooni AF, Khalili N, Liu X, et al. The Brain Tumor Segmentation (BraTS) Challenge 2023: Focus on Pediatrics (CBTN-CONNECT-DIPGR-ASNR-MICCAI BraTS-PEDs). ArXiv. 2024;arXiv:2305.17033v7.

Adewole M, Rudie JD, Gbdamosi A, et al. The Brain Tumor Segmentation (BraTS) Challenge 2023: Glioma Segmentation in Sub-Saharan Africa Patient Population (BraTS-Africa). ArXiv. 2023;arXiv:2305.19369v1.

Anazodo UC, Adewole M, Dako F. AI for population and global health in radiology. Radiol Artif Intell. 2022;4:e220107. PMID: 35923372

***Deng***

Kim MM, Parmar HA, Aryal MP, et al. Developing a pipeline for multiparametric MRI-guided radiation therapy: initial results from a phase II clinical trial in newly diagnosed glioblastoma. Tomography. 2019;5:118-126. PMID: 30854449

Kooreman ES, van Houdt PJ, Nowee ME, et al. Feasibility and accuracy of quantitative imaging on a 1.5 T MR-linear accelerator. Radiother Oncol. 2019;133:156-162. PMID: 30935572

Kerkmeijer LGW, Groen VH, Pos FJ, et al. Focal boost to the intraprostatic tumor in external beam radiotherapy for patients with localized prostate cancer: results from the FLAME randomized phase III trial. J Clin Oncol. 2021;39:787-796. PMID: 33471548

Mierzwa ML, Aryal M, Lee C, et al. Randomized phase II study of physiologic MRI-directed adaptive radiation boost in poor prognosis head and neck cancer. Clin Cancer Res. 2022;28:5049-5057. PMID: 36107219

Shoobridge AS, Baines JA. Evaluation of MU2net as an online secondary dose check for MR guided radiation therapy with the Elekta unity MR linac. Phys Eng Sci Med. 2022;45:429-441.

Kishan AU, Ma TM, Lamb JM, et al. Magnetic resonance imaging-guided vs computed tomography-guided stereotactic body radiotherapy for prostate cancer: The MIRAGE randomized clinical trial. JAMA Oncol. 2023;9:365-373. PMID: 35380366

Maas JA, McDonald AM, Cardan RA, et al. Characterization of photon intensity modulated radiation therapy robustness in patients with prostate cancer as a proposed benchmark for proton therapy robustness evaluation. Pract Radiat Oncol. 2024;14:e68-e74. PMID: 37748679

***Doneva***

Gazelle GS, Kessler L, Lee DW, et al. A framework for assessing the value of diagnostic imaging in the era of comparative effectiveness research. Radiology. 2011;261:692-8. PMID: 22095993

Yaman B, Hosseini SAH, Moeller S, Ellermann J, Uğurbil K, Akçakaya M. Self-supervised learning of physics-guided reconstruction neural networks without fully sampled reference data. Magn Reson Med. 2020;84:3172-3191. PMID: 32614100

Yoo J, Jin KH, Gupta H, Yerly J, Stuber M, Unser M. Time-dependent deep image prior for dynamic MRI. IEEE Trans Med Imaging. 2021;40:3337-3348. PMID: 34043506

Hammernik K, Küstner T, Yaman B, Huang Z, Rueckert D, Knoll F, Akçakaya M. Physics-driven deep learning for computational magnetic resonance imaging: combining physics and machine learning for improved medical imaging. IEEE Signal Process Mag. 2023 Jan;40:98-114. PMID: 37304755

Terzis R, Dratsch T, Hahnfeldt R, et al. Five-minute knee MRI: An AI-based super resolution reconstruction approach for compressed sensing. A validation study on healthy volunteers. Eur J Radiol. 2024;175:111418. PMID: 38490130

Harder FN, Weiss K, Amiel T, Peeters JM, et al. Prospectively accelerated t2-weighted imaging of the prostate by combining compressed sense and deep learning in patients with histologically proven prostate cancer. Cancers (Basel). 2022;14:5741. PMID: 36497223

Wang M, Ma Y, Li L, et al. Compressed sensitivity encoding artificial intelligence accelerates brain metastasis imaging by optimizing image quality and reducing scan time. AJNR Am J Neuroradiol. 2024;45:444-452. PMID: 38485196

***El Homsi***

Kusters M, Marijnen CA, van de Velde CJ, Rutten HJ, Lahaye MJ, Kim JH, Beets-Tan RG, Beets GL. Patterns of local recurrence in rectal cancer; a study of the Dutch TME trial. Eur J Surg Oncol. 2010;36:470-6. PMID: 20096534

Smith JJ, Chow OS, Gollub MJ, et al. Organ Preservation in Rectal Adenocarcinoma: a phase II randomized controlled trial evaluating 3-year disease-free survival in patients with locally advanced rectal cancer treated with chemoradiation plus induction or consolidation chemotherapy, and total mesorectal excision or nonoperative management. *BMC Cancer*. 2015;15:767. PMID: 26497495

Martens MH, van Heeswijk MM, van den Broek JJ, et al. Prospective, multicenter validation study of magnetic resonance volumetry for response assessment after preoperative chemoradiation in rectal cancer: can the results in the literature be reproduced? Int J Radiat Oncol Biol Phys. 2015;93:1005-1014. PMID: 26581139

Siddiqui MR, Gormly KL, Bhoday J, et al. Interobserver agreement of radiologists assessing the response of rectal cancers to preoperative chemoradiation using the MRI tumour regression grading (mrTRG). Clin Radiol. 2016;71:854-862. PMID: 27381221

Smith JJ, Strombom P, Chow OS, et al. Assessment of a watch-and-wait strategy for rectal cancer in patients with a complete response after neoadjuvant therapy. JAMA Oncol. 2019;5:e185896. PMID: 30629084

NCCN Clinical Practice Guidelines in Oncology. Rectal Cancer. Version 4.2024 .<https://www.nccn.org/guidelines/guidelines-detail?category=1&id=1461> [accessed May 5, 2025]

Jayaprakasam VS, Javed-Tayyab S, Gangai N, et al. Does microenema administration improve the quality of DWI sequences in rectal MRI? Abdom Radiol (NY). 2021;46:858-866. PMID: 32926212

Tan JJ, Carten RV, Babiker A, Abulafi M, Lord AC, Brown G. Prognostic importance of mri-detected extramural venous invasion in rectal cancer: a literature review and systematic meta-analysis. Int J Radiat Oncol Biol Phys. 2021;111:385-394. PMID: 34119593

Brouwer NPM, Lord AC, Terlizzo M, Bateman AC, West NP, Goldin R, Martinez A, Wong NACS, Novelli M, Nagtegaal ID, Brown G. Interobserver variation in the classification of tumor deposits in rectal cancer-is the use of histopathological characteristics the way to go? Virchows Arch. 2021;479(6):1111-1118. PMID: 34480612

Lord AC, D'Souza N, Shaw A, Rokan Z, Moran B, Abulafi M, Rasheed S, Chandramohan A, Corr A, Chau I, Brown G. MRI-diagnosed tumor deposits and EMVI status have superior prognostic accuracy to current clinical TNM staging in rectal cancer. Ann Surg. 2022;276(2):334-344. PMID: 32941279

Amin MB, Greene FL, Edge SB, et al. The Eighth Edition AJCC Cancer Staging Manual: Continuing to build a bridge from a population-based to a more "personalized" approach to cancer staging. CA Cancer J Clin. 2017;67:93-99. PMID: 28094848

Beets-Tan RGH, Lambregts DMJ, Maas M,et al. Magnetic resonance imaging for clinical management of rectal cancer: Updated recommendations from the 2016 European Society of Gastrointestinal and Abdominal Radiology (ESGAR) consensus meeting. Eur Radiol. 2018;28:1465-1475. PMID: 29043428

Ogura A, Konishi T, Cunningham C, et al. Neoadjuvant (Chemo)radiotherapy With Total Mesorectal Excision Only Is Not Sufficient to Prevent Lateral Local Recurrence in Enlarged Nodes: Results of the Multicenter Lateral Node Study of Patients With Low cT3/4 Rectal Cancer. J Clin Oncol. 2019;37:33-43. PMID: 30403572

Gollub MJ, Das JP, Bates DDB, et al. Rectal cancer with complete endoscopic response after neoadjuvant therapy: what is the meaning of a positive MRI? Eur Radiol. 2021;31:4731-4738. PMID: 33449186

Schaap DP, Boogerd LSF, Konishi T, et al. Rectal cancer lateral lymph nodes: multicentre study of the impact of obturator and internal iliac nodes on oncological outcomes. Br J Surg. 2021;108:205-213. PMID: 33711144

Garcia-Aguilar J, Patil S, Gollub MJ, et al. Organ preservation in patients with rectal adenocarcinoma treated with total neoadjuvant therapy. J Clin Oncol. 2022;40:2546-2556. PMID: 35483010

Yuval JB, Thompson HM, Firat C, et al. MRI at restaging after neoadjuvant therapy for rectal cancer overestimates circumferential resection margin proximity as determined by comparison with whole-mount pathology. Dis Colon Rectum. 2022;65:489-496. PMID: 34803147

Lee S, Kassam Z, Baheti AD, et al. Rectal cancer lexicon 2023 revised and updated consensus statement from the Society of Abdominal Radiology colorectal and anal cancer disease-focused panel. Abdom Radiol (NY). 2023;48:2792-2806. PMID: 37145311

Thompson HM, Omer DM, Lin S, et al. Organ preservation and survival by clinical response grade in patients with rectal cancer treated with total neoadjuvant therapy: a secondary analysis of the OPRA randomized clinical trial. JAMA Netw Open. 2024;7:e2350903. PMID: 38194231

***Greer***

Vanderby SA, Babyn PS, Carter MW, Jewell SM, McKeever PD. Effect of anesthesia and sedation on pediatric MR imaging patient flow. Radiology. 2010;256:229-237. PMID: 20505061

Carter AJ, Greer ML, Gray SE, Ware RS. Mock MRI: reducing the need for anaesthesia in children. Pediatr Radiol. 2010;40:1368-1374. PMID: 20186541

McGuirt D. Alternatives to sedation and general anesthesia in pediatric magnetic resonance imaging: a literature review. Radiol Technol. 2016;88:18-26. PMID: 27601689

Jaimes C, Gee MS. Strategies to minimize sedation in pediatric body magnetic resonance imaging. Pediatr Radiol. 2016;46:916-927. PMID: 27229508

Strouse PJ, Trout AT, Offiah AC. Editors' notebook: what is 'pediatric'? Pediatr Radiol. 2022;52:2241-2242. PMID: 36018347

Kraus MS, Yousef AA, Cote SL, Greer MC. Improving protocols for whole-body magnetic resonance imaging: oncological and inflammatory applications. Pediatr Radiol. 2023;53:1420-1442. PMID: 35982340

Uffman JC, Tumin D, Raman V, et al. MRI utilization and the associated use of sedation and anesthesia in a pediatric ACO. J Am Coll Radiol. 2017;14:924-930. PMID: 28325486

Kozak BM, Jaimes C, Kirsch J, Gee MS. MRI techniques to decrease imaging times in children. Radiographics. 2020;40:485-502. PMID: 32031912

Atun R, Bhakta N, Denburg A, et al. Sustainable care for children with cancer: a Lancet Oncology Commission. Lancet Oncol. 2020;21:e185-e224. PMID: 32240612

Artunduaga M, Liu CA, Morin CE, et al. Safety challenges related to the use of sedation and general anesthesia in pediatric patients undergoing magnetic resonance imaging examinations. Pediatr Radiol. 2021;51:724-735. PMID: 33860861

Machado-Rivas F, Leitman E, Jaimes C, et al. Predictors of anesthetic exposure in pediatric MRI. AJR Am J Roentgenol. 2021;216:799-805. PMID: 32755164

Sammer MBK, Akbari YS, Barth RA, et al. Use of artificial intelligence in radiology: impact on pediatric patients, a white paper from the ACR pediatric AI workgroup. J Am Coll Radiol. 2023;20:730-737. PMID: 37498259

Gallo-Bernal S, Peña-Trujillo V, Gee MS. Dual-energy computed tomography: pediatric considerations. Pediatr Radiol. 2024;54:2112-2126. PMID: 39470784

Ata NA, Trout AT, Dillman JR, Tkach JA, Ayyala RS. Technical and diagnostic performance of rapid MRI for evaluation of appendicitis in a pediatric emergency department. Acad Radiol. 2024;31:1102-1110. PMID: 37863782

[https://www.wfpiweb.org/Resources/Modalities/MRIProtocols.aspx​](https://www.wfpiweb.org/Resources/Modalities/MRIProtocols.aspx%E2%80%8B)

​

<https://www.sickkids.ca/en/care-services/clinical-departments/diagnostic-interventional-radiology/wags/>

<https://www.acr.org/Data-Science-and-Informatics/Informatics/pediatric-radiology-ai-resources>

***Guidon***

Taso M, Zhao L, Guidon A, Litwiller DV, Alsop DC. Volumetric abdominal perfusion measurement using a pseudo-randomly sampled 3D fast-spin-echo (FSE) arterial spin labeling (ASL) sequence and compressed sensing reconstruction. Magn Reson Med. 2019;82:680-692. PMID: 30953396

Pickhardt PJ. Abdominal imaging in the coming decades: better, faster, safer, and cheaper? Radiology. 2023 307:e222551. PMID: 36916892

Yoon JH, Lee JE, Park SH, Park JY, Kim JH, Lee JM. Comparison of image quality and lesion conspicuity between conventional and deep learning reconstruction in gadoxetic acid-enhanced liver MRI. Insights Imaging. 2024;15:257. PMID: 39466542

***Guimaraes and Wyatt***

Hanahan D, Weinberg RA. Hallmarks of cancer: the next generation. Cell. 2011;144:646-74. PMID: 21376230

Hamilton JI, Jiang Y, Chen Y, et al. MR fingerprinting for rapid quantification of myocardial T1, T2, and proton spin density. Magn Reson Med. 2017;77:1446-1458. PMID: 27038043

Tirkes T, Lin C, Cui E, Deng Y, Territo PR, Sandrasegaran K, Akisik F. Quantitative MR evaluation of chronic pancreatitis: extracellular volume fraction and MR relaxometry. AJR Am J Roentgenol. 2018;210:533-542. PMID: 29336598

Panda A, Obmann VC, Lo WC, et al. MR fingerprinting and ADC mapping for characterization of lesions in the transition zone of the prostate gland. Radiology. 2019;292:685-694. PMID: 31335285

Ostenson J, Damon BM, Welch EB. MR fingerprinting with simultaneous T1, T2, and fat signal fraction estimation with integrated B0 correction reduces bias in water T1 and T2 estimates. Magn Reson Imaging. 2019;60:7-19. PMID: 30910696

Jaubert O, Arrieta C, Cruz G, et al. Multi-parametric liver tissue characterization using MR fingerprinting: Simultaneous T1 , T2 , T2*, and fat fraction mapping. Magn Reson Med. 2020;84:2625-2635. PMID: 32406125

Choi MH, Lee SW, Kim HG, Kim JY, Oh SW, Han D, Kim DH. 3D MR fingerprinting (MRF) for simultaneous T1 and T2 quantification of the bone metastasis: Initial validation in prostate cancer patients. Eur J Radiol. 2021;144:109990. PMID: 34638082

Huang SS, Boyacioglu R, Bolding R, MacAskill C, Chen Y, Griswold MA. Free-breathing abdominal magnetic resonance fingerprinting using a pilot tone navigator. J Magn Reson Imaging. 2021;54:1138-1151. PMID: 33949741

Starekova J, Hernando D, Pickhardt PJ, Reeder SB. Quantification of liver fat content with CT and MRI: state of the art. Radiology. 2021;301:250-262. PMID: 34546125

Tirkes T, Yadav D, Conwell DL, et al. Quantitative MRI of chronic pancreatitis: results from a multi-institutional prospective study, magnetic resonance imaging as a non-invasive method for assessment of pancreatic fibrosis (MINIMAP). Abdom Radiol (NY). 2022;47:3792-3805. PMID: 36038644

Sharafi A, Zibetti MVW, Chang G, Cloos MA, Regatte RR. Simultaneous bilateral T1 , T2 , and T1ρ relaxation mapping of the hip joint with magnetic resonance fingerprinting. NMR Biomed. 2022;35:e4651. PMID: 34825750

Velasco C, Cruz G, Jaubert O, Lavin B, Botnar RM, Prieto C. Simultaneous comprehensive liver T1 , T2 , T2∗ , T1ρ , and fat fraction characterization with MR fingerprinting. Magn Reson Med. 2022;87:1980-1991. PMID: 34792212

Cao X, Liao C, Iyer SS, et al. Optimized multi-axis spiral projection MR fingerprinting with subspace reconstruction for rapid whole-brain high-isotropic-resolution quantitative imaging. Magn Reson Med. 2022;88:133-150. PMID: 35199877

Lee YS, Choi MH, Lee YJ, Han D, Kim DH. Magnetic resonance fingerprinting in prostate cancer before and after contrast enhancement. Br J Radiol. 2022;1;95:20210479. PMID: 34415785

​de Oliveira Correia ET, Qiao PL, Griswold MA, Chen Y, Bittencourt LK. Magnetic resonance fingerprinting based comprehensive quantification of T1 and T2 values of the background prostate peripheral zone: Correlation with clinical and demographic features. Eur J Radiol. 2023;164:110883. PMID: 37209463

Fujita S, Sano K, Cruz G, et al. MR Fingerprinting for liver tissue characterization: A Histopathologic Correlation Study. Radiology. 2023;306:150-159. PMID: 36040337

Liu C, Li T, Cao P, Hui ES, et al. Respiratory-correlated 4-dimensional magnetic resonance fingerprinting for liver cancer radiation therapy motion Management. Int J Radiat Oncol Biol Phys. 2023;117:493-504. PMID: 37116591

Reeder SB, Yokoo T, França M, et al. Quantification of liver iron overload with MRI: review and guidelines from the ESGAR and SAR. Radiology. 2023;307:e221856. PMID: 36809220

MacAskill CJ, Kretzler ME, Parsons A, et al. Multimodal Magnetic Resonance Imaging Assessments of Kidney Disease Severity in Autosomal Recessive Polycystic Kidney Disease. Kidney Int Rep. 2024;9:3592-3595. PMID: 39698366

Dupuis A, Chen Y, Hansen M, Chow K, et al. Quantifying 3D MR fingerprinting (3D-MRF) reproducibility across subjects, sessions, and scanners automatically using MNI atlases. Magn Reson Med. 2024;91:2074-2088. PMID: 38192239

***Gurney-Champion***

Golkov V, Dosovitskiy A, Sperl JI, et al. q-space deep learning: twelve-fold shorter and model-free diffusion MRI scans. IEEE Trans Med Imaging. 2016;35:1344–1351. PMID: 27071165

Bertleff M, Domsch S, Weingärtner S, et al. Diffusion parameter mapping with the combined intravoxel incoherent motion and kurtosis model using artificial neural networks at 3 T. NMR Biomed. 2017;30:1-11. PMID: 28960549

Hubertus S, Thomas S, Cho J, Zhang S, Wang Y, Schad LR. Using an artificial neural network for fast mapping of the oxygen extraction fraction with combined QSM and quantitative BOLD. Magn Reson Med. 2019;82:2199-2211. PMID: 31273828

Bliesener Y, Acharya J, Nayak KS. Efficient DCE-MRI parameter and uncertainty estimation using a neural network. IEEE Trans Med Imaging. 2020;39:1712-1723. PMID: 31794389

Zou J, Balter Jm, Cao Y. Estimation of pharmacokinetic parameters from DCE-MRI by extracting long and short time-dependent features using an LSTM network. Med Phys. 2020;47:3447–3457. PMID: 32379942

Barbieri, S, Gurney-Champion OJ, Klaassen R, Thoeny HC. Deep learning how to fit an intravoxel incoherent motion model to diffusion-weighted MRI. Magn Reson Med. 2020;83:312-321. PMID: 31389081

Kaandorp MPT, Barbieri S, Klaassen R, et al. Improved unsupervised physics-informed deep learning for intravoxel incoherent motion modeling and evaluation in pancreatic cancer patients. Magn Reson Med. 2021;86:2250-2265. PMID: 34105184

Zhang C, Karkalousos D, Bazin PL, et al. A unified model for reconstruction and R2* mapping of accelerated 7T data using the quantitative recurrent inference machine. Neuroimage. 2022;264:119680. PMID: 36240989

Ottens T, Barbieri S, Orton MR, et al. Deep learning DCE-MRI parameter estimation: Application in pancreatic cancer. Med Image Anal. 2022;80:102512. PMID: 35709559

Vasylechko SD, Warfield SK, Afacan O, Kurugol S. Self-supervised IVIM DWI parameter estimation with a physics based forward model. Magn Reson Med. 2022;87:904-914. PMID: 34687065

***Harmath***

Ho S, Lau WY, Leung TW, Johnson PJ. Internal radiation therapy for patients with primary or metastatic hepatic cancer: a review. Cancer. 1998;83:1894-1907.​ PMID: 9806647

Olsen CC, Welsh J, Kavanagh BD, et al. Microscopic and macroscopic tumor and parenchymal effects of liver stereotactic body radiotherapy. Int J Radiat Oncol Biol Phys. 2009;73:1414-1424. PMID: 18990508

Kis B, El-Haddad G, Sheth RA, et al. Liver-directed therapies for hepatocellular carcinoma and intrahepatic cholangiocarcinoma. Cancer Control. 2017;24:107327481772924. PMID: 28975829

Semaan S, Makkar J, Lewis S, Chatterji M, Kim E, Taouli B. Imaging of hepatocellular carcinoma response after Y90 radioembolization. AJR Am J Roentgenol. 2017;209(5):W263-W276. PMID: 29072955

Takamatsu S, Kozaka K, Kobayashi S, et al. Pathology and images of radiation-induced hepatitis: a review article. Jpn J Radiol. 2018;36:241-256. PMID: 29508261

Aslam A, Do RKG, Kambadakone A, et al. Hepatocellular carcinoma Liver Imaging Reporting and Data Systems treatment response assessment: Lessons learned and future directions. World J Hepatol. 2020;12:738-753. PMID: 33200013

Do RK, Mendiratta-Lala M. LI-RADS Version 2018 treatment response algorithm: the evidence is accumulating. Radiology. 2020;294:327-328. PMID: 31846402

Salem R, Lewandowski RJ, Mulcahy MF, et al. Radioembolization for hepatocellular carcinoma using Yttrium-90 microspheres: a comprehensive report of long-term outcomes. Gastroenterology. 2010;138:52-64. PMID: 19766639

Mendiratta-Lala M, Masch WR, Shampain K, et al. MRI assessment of hepatocellular carcinoma after local-regional therapy: a comprehensive review. Radiol Imaging Cancer. 2020;2:e190024. PMID: 33778692

Do RK, Mendiratta-Lala M. Moving away from uncertainty: a potential role for ancillary features in li-rads treatment response. Radiology. 2020;296:562-563. PMID: 32697166

Benson AB, D'Angelica MI, Abbott DE, et al. Hepatobiliary Cancers, Version 2.2021, NCCN Clinical Practice Guidelines in Oncology. J Natl Compr Canc Netw. 2021;19:541-565. PMID: 34030131

​

Shampain KL, Hackett CE, Towfighi S, et al. SBRT for HCC: Overview of technique and treatment response assessment. Abdom Radiol N Y. 2021;46:3615-3624. PMID: 33963419

Apisarnthanarax S, Barry A, Cao M, et al. External Beam Radiation Therapy for Primary Liver Cancers: An ASTRO Clinical Practice Guideline. Pract Radiat Oncol. 2022;12:28-51. PMID: 34688956

Lucatelli P, Guiu B. 2022 Update of BCLC treatment algorithm of hcc: what's new for interventional radiologists? Cardiovasc Intervent Radiol. 2022;45:275-276. PMID: 35088139

Navin PJ, Olson MC, Mendiratta-Lala M, Hallemeier CL, Torbenson MS, Venkatesh SK. Imaging features in the liver after stereotactic body radiation therapy. RadioGraphics. 2022;42:2131-2148. PMID: 36240077

American College of Radiology. LI-RADS treatment response, available at:<https://www.acr.org/Clinical-Resources/Clinical-Tools-and-Reference/Reporting-and-Data-Systems/LI-RADS>

Villalobos A, Arndt L, Cheng B, et al. Yttrium-90 Radiation segmentectomy of hepatocellular carcinoma: a comparative study of the effectiveness, safety, and dosimetry of glass-based versus resin-based microspheres. J Vasc Interv Radiol JVIR. 2023;34:1226-1234. PMID: 36958669

Patel R, Aslam A, Parikh ND, et al. Updates on LI-RADS treatment response criteria for hepatocellular carcinoma: focusing on MRI. J Magn Reson Imaging JMRI. 2023;57:1641-1654. PMID: 36872608

Soon GST, Yasir S, Wu TT, et al. unique morphologic findings in the liver after stereotactic radiation for cholangiocarcinoma. Am J Surg Pathol. 2023;47:792-800. PMID: 37204143

Inchingolo R, Cortese F, Pisani AR, et al. Selective internal radiation therapy segmentectomy: A new minimally invasive curative option for primary liver malignancies? World J Gastroenterol. 2024;30:2379-2386. PMID: 38764771

Deodato F, Pezzulla D, Cilla S, Romano C, Ferro M, Galietta E, Lancellotta V, Morganti AG, Macchia G. Stereotactic radiosurgery with volumetric modulated arc radiotherapy: Final results of a multi-arm phase I trial (DESTROY-2). Clin Oncol (R Coll Radiol). 2024;36:632-641. PMID: 38971684

Rodríguez MCR, Chen-Zhao X, Hernando O, et al. SBRT-SG-01: final results of a prospective multicenter study on stereotactic body radiotherapy for liver metastases. Clin Transl Oncol Off Publ Fed Span Oncol Soc Natl Cancer Inst Mex. 2024;26:1790-1797. PMID: 38431539

***Hirsch***

Uecker M, Zhang S, Voit D, Karaus A, Merboldt KD, Frahm J. Real-time MRI at a resolution of 20 ms. NMR Biomed. 2010;23:986–994. PMID: 20799371

Zhang S, Joseph AA, Voit D, et al. Real-time magnetic resonance imaging of cardiac function and flow - recent progress. Quant Imaging Med Surg. 2014;4:313–329. PMID: 25392819

Schaetz S, Voit D, Frahm J, Uecker M. Accelerated computing in magnetic resonance imaging: real-time imaging using nonlinear inverse reconstruction. Comput Math Methods Med. 2017;2017:3527269. PMID: 29463984

Frahm J, Voit D, Uecker M. Real-time magnetic resonance imaging: radial gradient-echo sequences with nonlinear inverse reconstruction. Invest Radiol. 2019;54:757–766. PMID: 31261294

Röwer LM, Radke KL, Hußmann J, et al. Comparison of cardiac volumetry using real-time MRI during free-breathing with standard cine MRI during breath-hold in children. Pediatr Radiol. 2022;52:1462–1475. PMID: 35353211

Sorge I, Hirsch FW, Voit D, et al. Decreased need for anesthesia during ultra-fast cranial MRI in young children: one-year summary. Rofo. 2022;194:192–198. PMID: 34644801

Laubrock K, von Loesch T, Steinmetz M, Lotz J, Frahm J, Uecker M, Unterberg-Buchwald C. Imaging of arrhythmia: real-time cardiac magnetic resonance imaging in atrial fibrillation. Eur J Radiol Open. 2022;9:100404. PMID: 35265735

Liszewski MC, Pierluigi C, Winant AJ, Lee EY. Lung and large airway imaging: magnetic resonance imaging versus computed tomography. Pediatr Radiol. 2022;52:1814–1825. PMID: 35570212

Hellwig SJ, Iltis PW, Joseph AA, Voit D, Frahm J, Schoonderwaldt E, Altenmüller E. Tongue involvement in embouchure dystonia: new piloting results using real-time MRI of trumpet players. J Clin Mov Disord. 2019;6:5. PMID: 31754440

Krohn S, Joseph AA, Voit D, Michaelis T, Merboldt KD, Buergers R, Frahm J. Multi-slice real-time MRI of temporomandibular joint dynamics. Dentomaxillofac Radiol. 2019;48:20180162. PMID: 30028188

Seif Amir Hosseini A, Uhlig J, Streit U, et al. Biggemann. Real-time MRI for dynamic assessment of gastroesophageal reflux disease: comparison to pH-metry and impedance. Eur J Radiol. 2020;125:108856. PMID: 32062571

Gräfe D, Lacher M, Martynov I, et al. Pectus excavatum in motion: dynamic evaluation using real-time MRI. Eur Radiol. 2023;33:2128-2135. PMID: 36307555

Hirsch FW, Frahm J, Sorge I, et al. Real-time MRI: a new tool of radiologic imaging in small children. Eur J Pediatr. 2023;182(8):3405-3417. doi:10.1007/s00431-023-04996-0. PMID: 37249681

***Kang***

Jørgensen T, Jensen KH. Polyps in the gallbladder. A prevalence study. Scand J Gastroenterol. 1990;25:281-286. PMID: 2320947

Nguyen XV, Davies L, Eastwood JD, Hoang JK. Extrapulmonary findings and malignancies in participants screened with chest CT in the national lung screening trial. J Am Coll Radiol. 2017;14:324-330. PMID: 28259326

Cohen JD, Li L, Wang Y, Thoburn C, Afsari B, Danilova L, et al. Detection and localization of surgically resectable cancers with a multi-analyte blood test. Science. 2018;359:926-930.​ PMID: 29348365

Lennon AM, Buchanan AH, Kinde I, et al. Feasibility of blood testing combined with PET-CT to screen for cancer and guide intervention. Science. 2020;369(6499).​ PMID: 32345712

Kang SK, Scherer LD, Megibow AJ, et al. A randomized study of patient risk perception for incidental renal findings on diagnostic imaging tests. AJR Am J Roentgenol. 2018;210:369-375. PMID: 29140116

Mahesh M, Ansari AJ, Mettler, Jr, FA. Patient exposure from radiologic and nuclear medicine procedures in the United States and Worldwide: 2009–2018. Radiology 2023;307:e22126. ​PMID: 36511806

​

Schrag D, Beer TM, McDonnell CH, et al. Blood-based tests for multi cancer early detection (PATHFINDER): a prospective cohort study. Lancet. 2023;402:1251-1260.​ PMID: 37805216

​

Kim DH, Kim SW, Basurrah MA, Lee J, Hwang SH. Diagnostic performance of six ultrasound risk stratification systems for thyroid nodules: a systematic review and network meta-analysis. AJR Am J Roentgenol. 2023;220:791-803.​ PMID: 36725367

Nicholson BD, Oke J, Virdee PS, Harris DA, O'Doherty C, Park JE, et al. Multi-cancer early detection test in symptomatic patients referred for cancer investigation in England and Wales (SYMPLIFY): a large-scale, observational cohort study. Lancet Oncol. 2023;24:733-743.​ PMID: 3352875

Lennon AM, Buchanan AH, Rego SP, et al. Outcomes following a false positive multi-cancer early detection (MCED) test: Results from DETECT-A, the first large, prospective, interventional MCED study. Cancer Prev Res (Phila). 2024. doi: 10.1158/1940-6207.CAPR-23-0451. PMID: 38705577

***Küstner***

Küstner T, Liebgott A, Mauch L, Martirosian P, Bamberg F, Nikolaou K, Yang B, Schick F, Gatidis S. Automated reference-free detection of motion artifacts in magnetic resonance images. MAGMA. 2018;31:243-256. PMID: 28932991

Küstner T, Armanious K, Yang J, Yang B, Schick F, Gatidis S. Retrospective correction of motion-affected MR images using deep learning frameworks. Magn Reson Med. 2019;82:1527-1540. PMID: 31081955

Haskell MW, Cauley SF, Bilgic B, et al. Network accelerated motion estimation and reduction (NAMER): Convolutional neural network guided retrospective motion correction using a separable motion model. Magn Reson Med. 2019;82:1452-1461. PMID: 31045278

Armanious K, Jiang C, Fischer M, et al. MedGAN: Medical image translation using GANs. Comput Med Imaging Graph. 2020;79:101684. PMID: 31812132

***Mächann***

Berg D, Youdim MB. Role of iron in neurodegenerative disorders. Top Magn Reson Imaging. 2006;17:5-17. PMID: 17179893

Fox CS, Massaro JM, Hoffmann U, et al. Abdominal visceral and subcutaneous adipose tissue compartments: association with metabolic risk factors in the Framingham Heart Study. Circulation. 2007;116:39-48. PMID: 17576866

Machann J, Thamer C, Stefan N, et al. Follow-up whole-body assessment of adipose tissue compartments during a lifestyle intervention in a large cohort at increased risk for Type 2 diabetes. Radiology. 2010;257:353-363. PMID: 20713612

Fleming RE, Ponka P. Iron overload in human disease. N Engl J Med. 2012;366:348-359. PMID: 22276824

Bamberg F, Hetterich H, Rospleszcz S, et al. Subclinical disease burden as assessed by whole-body MRI in subjects with prediabetes, subjects with diabetes, and normal control subjects from the general population: the KORA-MRI study. Diabetes. 2017;66:158-169. PMID: 27999110

Machann J, Stefan N, Wagner R, et al. Intra- and interindividual variability of fatty acid unsaturation in six different human adipose tissue compartments assessed by 1 HMRS in vivo at 3 T. NMR Biomed. 2017;30. doi: 10.1002/nbm.3744. PMID: 28543814

Liu S, Buch S, Chen Y, Choi HS, et al. Susceptibility-weighted imaging: current status and future directions. NMR Biomed. 2017;30. doi: 10.1002/nbm.3552. PMID: 27192086

Linge J, Borga M, West J, et al. Body composition profiling in the UK Biobank imaging study. Obesity (Silver Spring). 2018;26:1785-1795. PMID: 29785727

Borga M, West J, Bell JD, Harvey NC, Romu T, Heymsfield SB, Leinhard OD. Advanced body composition assessment: from body mass index to body composition profiling. J Investig Med. 2018;66:1-9. PMID: 29581385

Jhaveri KS, Kannengiesser SAR, Ward R, Kuo K, Sussman MS. Prospective evaluation of an R2* method for assessing liver iron concentration (LIC) against FerriScan: derivation of the calibration curve and characterization of the nature and source of uncertainty in the relationship. J Magn Reson Imaging. 2019;49:1467-1474. PMID: 30291649

Ghassaban K, Liu S, Jiang C, Haacke EM. Quantifying iron content in magnetic resonance imaging. Neuroimage. 2019;187:77-92. PMID: 29702183

Neeland IJ, Ross R, Després JP, et al. Visceral and ectopic fat, atherosclerosis, and cardiometabolic disease: a position statement. Lancet Diabetes Endocrinol. 2019;7:715–725. PMID: 31301983

Rado SD, Lorbeer R, Gatidis S, et al. MRI-based assessment and characterization of epicardial and paracardial fat depots in the context of impaired glucose metabolism and subclinical left-ventricular alterations. Br J Radiol. 2019;92:20180562. PMID: 30633543

Schneider M, Janas G, Lugauer F, et al. Accurate fatty acid composition estimation of adipose tissue in the abdomen based on bipolar multi-echo MRI. Magn Reson Med. 2019;81:2330-2346. PMID: 30368904

Roumans KHM, Lindeboom L, Veeraiah P, et al. Hepatic saturated fatty acid fraction is associated with de novo lipogenesis and hepatic insulin resistance. Nat Commun. 2020;11:1891. PMID: 32312974

Hernando D, Cook RJ, Qazi N, Longhurst CA, Diamond CA, Reeder SB. Complex confounder-corrected R2* mapping for liver iron quantification with MRI. Eur Radiol. 2021;31:264-275. PMID: 32785766

Wagner R, Heni M, Tabák AG, et al. Pathophysiology-based sub phenotyping of individuals at elevated risk for type 2 diabetes. Nat Med. 2021;27:49-57. PMID: 33398163

Kiefer LS, Fabian J, Rospleszcz S,et al. Distribution patterns of intra- and extramyocellular fat by magnetic resonance imaging in subjects with diabetes, prediabetes and normoglycemic controls. Diabetes Obes Metab. 2021;23:1868-1878. PMID: 33914415

Idilman IS, Yildiz AE, Karaosmanoglu AD, Ozmen MN, Akata D, Karcaaltincaba M. Proton density fat fraction: magnetic resonance imaging applications beyond the liver. Diagn Interv Radiol. 2022;28:83-91. PMID: 35142615

Haueise T, Schick F, Stefan N, et al. Analysis of volume and topography of adipose tissue in the trunk: results of MRI of 11,141 participants in the German National Cohort. Sci Adv. 2023;9:eadd0433. PMID: 37172093

***Margolis***

Niwa T, Ueno M, Ohkawa S, et al. Advanced pancreatic cancer: the use of the apparent diffusion coefficient to predict response to chemotherapy. Br J Radiol. 2009;82:28–34. PMID: 19095814

Amodeo S, Rosman AS, Desiato V, et al. MRI-based apparent diffusion coefficient for predicting pathologic response of rectal cancer after neoadjuvant therapy: systematic review and meta-analysis. AJR Am J Roentgenol. 2018;211:W205–W216. PMID: 30240291

Li Q, Wang D, Zhu X, Shen K, Xu F, Chen Y. Combination of renal apparent diffusion coefficient and renal parenchymal volume for better assessment of split renal function in chronic kidney disease. Eur J Radiol. 2018;108:194–200. PMID: 30396655

Lewis S, Peti S, Hectors SJ, et al. Volumetric quantitative histogram analysis using diffusion-weighted magnetic resonance imaging to differentiate HCC from other primary liver cancers. Abdom Radiol (NY). 2019;44:912–922. PMID: 30712136

Moraes MO, Roman DHH, Copetti J, et al. Effects of the addition of quantitative apparent diffusion coefficient data on the diagnostic performance of the PI-RADS v2 scoring system to detect clinically significant prostate cancer. World J Urol. 2020;38:981–991. PMID: 31175458

Meyer H-J, Wienke A, Surov A. Discrimination between clinical significant and insignificant prostate cancer with apparent diffusion coefficient - a systematic review and meta-analysis. BMC Cancer. 2020; 20:482. PMID: 32460795

Ao W, Bao X, Mao G, Yang G, Wang J, Hu J. Value of apparent diffusion coefficient for assessing preoperative T Staging of low rectal cancer and whether this is correlated with Ki-67 expression. Can Assoc Radiol J. 2020;71:5-11. PMID: 32063001

Yamamoto S, Yoshida S, Ishii C, et al. Metastatic diffusion volume based on apparent diffusion coefficient as a prognostic factor in castration-resistant prostate cancer. J Magn Reson Imaging. 2021;54:401–408. PMID: 33694240

Wang Y, Tadimalla S, Rai R, et al. Quantitative MRI: Defining repeatability, reproducibility and accuracy for prostate cancer imaging biomarker development. Magn Reson Imaging. 2021;77:169–179. PMID: 33388362

Duan S, Geng L, Lu F, et al. Utilization of the corticomedullary difference in magnetic resonance imaging-derived apparent diffusion coefficient for noninvasive assessment of chronic kidney disease in type 2 diabetes. Diabetes Metab Syndr. 2024;18:102693. PMID: 38373384

Rimola J, Fernandez-Clotet A, Capozzi N, et al. ADC values for detecting bowel inflammation and biologic therapy response in patients with crohn disease: A post hoc prospective trial analysis. AJR Am J Roentgenol. 2024;222:e2329639. PMID: 37584507

***Muglia***

Heidenreich A, Bellmunt J, Bolla M, et al. EAU guidelines on prostate cancer. part 1: screening, diagnosis, and treatment of clinically localised disease. Eur Urol. 2011;59:61-71. PMID: 21056534

Rosenkrantz AB, Kim S, Lim RP, et al. Prostate cancer localization using multiparametric MR imaging: comparison of Prostate Imaging Reporting and Data System (PI-RADS) and Likert scales. Radiology. 2013;269:482-492. PMID: 23788719

Wu LM, Xu JR, Gu HY, et al. Role of magnetic resonance imaging in the detection of local prostate cancer recurrence after external beam radiotherapy and radical prostatectomy. Clin Oncol (R Coll Radiol). 2013;25:252-64. PMID: 23313568

Mottet N, Bellmunt J, Bolla M, et al. EAU-ESTRO-SIOG Guidelines on Prostate cancer. part 1: screening, diagnosis, and local treatment with curative intent. Eur Urol. 2017;71:618-629. PMID: 27568654

Moore CM, Giganti F, Albertsen P, et al. Reporting magnetic resonance imaging in men on active surveillance for prostate cancer: The PRECISE recommendations-a report of a European school of oncology task force. Eur Urol. 2017;71:648-655. PMID: 27349615

Kasivisvanathan V, Rannikko AS, Borghi M, et al. MRI-targeted or standard biopsy for prostate-cancer diagnosis. N Engl J Med. 2018;378(19):1767-1777. PMID: 29552975

Padhani AR, Barentsz J, Villeirs G, et al. PI-RADS Steering Committee: The PI-RADS multiparametric mri and mri-directed biopsy pathway. Radiology. 2019;292:464-474. PMID: 31184561

Giganti F, Allen C, Emberton M, Moore CM, Kasivisvanathan V; PRECISION study group. Prostate Imaging Quality (PI-QUAL): A new quality control scoring system for multiparametric magnetic resonance imaging of the prostate from the PRECISION trial. Eur Urol Oncol. 2020;3:615-619. PMID: 32646850

Rajwa P, Pradere B, Quhal F, et al. Reliability of serial prostate magnetic resonance imaging to detect prostate cancer progression during active surveillance: a systematic review and meta-analysis. Eur Urol. 2021;80:549-563. PMID: 34020828

Brembilla G, Lavalle S, Parry T, et al. Impact of prostate imaging quality (PI-QUAL) score on the detection of clinically significant prostate cancer at biopsy. Eur J Radiol. 2023;164:110849. PMID: 37141845

de Rooij M, Allen C, Twilt JJ, et al. PI-QUAL version 2: an update of a standardised scoring system for the assessment of image quality of prostate MRI. Eur Radiol. 2024;34:7068-7079. PMID: 38787428

Franco PN, Frade-Santos S, García-Baizán A, et al. An MRI assessment of prostate cancer local recurrence using the PI-RR system: diagnostic accuracy, inter-observer reliability among readers with variable experience, and correlation with PSA values. Eur Radiol. 2024;34:1790-1803. PMID: 37646815

Light A, Mayor N, Cullen E, et al. The transatlantic recommendations for prostate gland evaluation with magnetic resonance imaging after focal therapy (TARGET): a systematic review and international consensus recommendations. Eur Urol. 2024;85:466-482. PMID: 38519280

Englman C, Maffei D, Allen C, et al. PRECISE Version 2: Updated recommendations for reporting prostate magnetic resonance imaging in patients on active surveillance for prostate cancer. Eur Urol. 2024;86:240-255. PMID: 38556436

Pausch AM, Filleböck V, Elsner C, Rupp NJ, Eberli D, Hötker AM. Ultra-fast biparametric MRI in prostate cancer assessment: diagnostic performance and image quality compared to conventional multiparametric MRI. Eur J Radiol Open. 2025;14:100635. PMID: 39906153

Esengur OT, Gelikman DG, Law YM, et al. Comparison of transatlantic recommendations for prostate gland evaluation with MRI after focal therapy (TARGET) and prostate imaging after focal ablation (PI-FAB) for detecting recurrent prostate cancer at prostate MRI. Acad Radiol. 2025;32:855-863. PMID: 39426916

***Nickel***

Hammernik K, Klatzer T, Kobler E, et al. Learning a variational network for reconstruction of accelerated MRI data. Magn Reson Med. 2018;79:3055-3071. PMID: 29115689

Schlemper J, Caballero J, Hajnal JV, Price AN, Rueckert D. A deep cascade of convolutional neural networks for dynamic MR image reconstruction. IEEE Trans Med Imaging. 2018;37:491-503. PMID: 29035212

Chen F, Taviani V, Malkiel I, et al. Variable-density single-shot fast spin-echo MRI with deep learning reconstruction by using variational networks. Radiology. 2018;289:366-373. PMID: 30040039

Yaman B, Hosseini SAH, Moeller S, Ellermann J, Uğurbil K, Akçakaya M. Self-supervised learning of physics-guided reconstruction neural networks without fully sampled reference data. Magn Reson Med. 2020;84:3172-3191. PMID: 32614100

Jafari R, Spincemaille P, Zhang J, et al. Deep neural network for water/fat separation: Supervised training, unsupervised training, and no training. Magn Reson Med. 2021;85:2263-2277. PMID: 33107127

Gadjimuradov F, Benkert T, Nickel MD, Maier A. Robust partial Fourier reconstruction for diffusion-weighted imaging using a recurrent convolutional neural network. Magn Reson Med. 2022;87:2018-2033. PMID: 34841550

Lin DJ, Walter SS, Fritz J. Artificial intelligence-driven ultra-fast superresolution MRI: 10-fold accelerated musculoskeletal turbo spin echo MRI within reach. Invest Radiol. 2023;58:28-42. PMID: 36355637

Ginocchio LA, Smereka PN, Tong A, et al. Accelerated T2-weighted MRI of the liver at 3 T using a single-shot technique with deep learning-based image reconstruction: impact on the image quality and lesion detection. Abdom Radiol (NY). 2023;48:282-290. PMID: 36171342

Bae SH, Hwang J, Hong SS, et al. Clinical feasibility of accelerated diffusion weighted imaging of the abdomen with deep learning reconstruction: Comparison with conventional diffusion weighted imaging. Eur J Radiol. 2022;154:110428. PMID: 35797791

Brendel JM, Jacoby J, Dehdab R, et al. Deep learning reconstruction for accelerated high-resolution upper abdominal MRI improves lesion detection without time penalty. Diagn Interv Imaging. 2025;106:85-92. PMID: 39567306

***Ohliger***

Langner T, Hedström A, Mörwald K, et al. Fully convolutional networks for automated segmentation of abdominal adipose tissue depots in multicenter water-fat MRI. Magn Reson Med. 2019;81:2736-2745. PMID: 30311704

Han S, Lee JM, Kim SW, Park S, Nickel MD, Yoon JH. Evaluation of HASTE T2 weighted image with reduced echo time for detecting focal liver lesions in patients at risk of developing hepatocellular carcinoma. Eur J Radiol. 2022;157:110588. PMID: 36345087

Boschheidgen M, Drewes L, Valentin B, et al. Use of deep learning-accelerated T2 TSE for prostate MRI: Comparison with and without hyoscine butylbromide admission. Magn Reson Imaging. 2025;118:110358. PMID: 39938669

Sharbatdaran A, Romano D, Teichman K, et al. Deep learning automation of kidney, liver, and spleen segmentation for organ volume measurements in autosomal dominant polycystic kidney disease. Tomography. 2022;8:1804-1819. PMID: 35894017

Rickmann AM, Senapati J, Kovalenko O, Peters A, Bamberg F, Wachinger C. AbdomenNet: deep neural network for abdominal organ segmentation in epidemiologic imaging studies. BMC Med Imaging. 2022;22:168. PMID: 36115938

Kanakaraj P, Ramadass K, Bao S, et al. Workflow integration of research AI tools into a hospital radiology rapid prototyping environment. J Digit Imaging. 2022;35:1023-1033. PMID: 35266088

***Otero and Serai***

<https://www.parametricmri.com/>

Serai SD, Elsingergy MM, Hartung EA, Otero HJ. Liver and spleen volume and stiffness in patients post-Fontan procedure and patients with ARPKD compared to normal controls. Clin Imaging. 2022;89:147-154. PMID: 35835018

Otero HJ, Calle-Toro JS, Maya CL, Darge K, Serai SD. DTI of the kidney in children: comparison between normal kidneys and those with ureteropelvic junction (UPJ) obstruction. MAGMA. 2020;33(1):63-71. PMID: 31845301

***Pagel***

Yankeelov TE, Luci JJ, Lepage M, et al. Quantitative pharmacokinetic analysis of DCE-MRI data without an arterial input function: a reference region model. Magn Reson Imaging. 2005;23:519-529. PMID: 15919597

Chen LQ, Howison CM, Jeffery JJ, Robey IF, Kuo PH, Pagel MD. Evaluations of extracellular pH within in vivo tumors using acidoCEST MRI. Magn Reson Med. 2014;72:1408-1417. PMID: 24281951

Chen LQ, Randtke EA, Jones KM, Moon BF, Howison CM, Pagel MD. Evaluations of tumor acidosis within in vivo tumor models using parametric maps generated with acido CEST MRI. Mol Imaging Biol. 2015;17:488-496. PMID: 25622809

Jones KM, Pollard AC, Pagel MD. Clinical applications of chemical exchange saturation transfer (CEST) MRI. J Magn Reson Imaging. 2018;47:11-27. PMID: 28792646

***Serkova***

Soylu FN, Peng Y, Jiang Y, et al. Seminal vesicle invasion in prostate cancer: evaluation by using multiparametric endorectal MR imaging. Radiology. 2013;267:797-806. PMID: 23440325

Yap TA, Arkenau HT, Camidge DR, et al. First-in-human phase I trial of two schedules of OSI-930, a novel multikinase inhibitor, incorporating translational proof-of-mechanism studies. Clin Cancer Res. 2013;19:909-919. PMID: 23403628

Armbruster M, Sourbron S, Haug A, et al. Evaluation of neuroendocrine liver metastases: a comparison of dynamic contrast-enhanced magnetic resonance imaging and positron emission tomography/computed tomography. Invest Radiol. 2014;49:7-14. PMID: 24002080

Fredrickson J, Serkova NJ, Wyatt SK, et al. Clinical translation of ferumoxytol-based vessel size imaging (VSI): Feasibility in a phase I oncology clinical trial population. Magn Reson Med. 2017;77:814-825. PMID: 26918893

Weiss J, Martirosian P, Notohamiprodjo M, et al. Implementation of a 5-minute magnetic resonance imaging screening protocol for prostate cancer in men with elevated prostate-specific antigen before biopsy. Invest Radiol. 2018;53:186-190. PMID: 29077588

Feng X, Chen X, Peng P, et al. Values of multiparametric and biparametric MRI in diagnosing clinically significant prostate cancer: a multivariate analysis. BMC Urol. 2024;24:40. PMID: 38365673

Li W, Le NN, Nadkarni R, et al. Tumor morphology for prediction of poor responses early in neoadjuvant chemotherapy for breast cancer: a multicenter retrospective study. Tomography. 2024;10:1832-1845. PMID: 39590943

***Razakamanantsoa***

Blazeby JM, Wilson L, Metcalfe C, Nicklin J, English R, Donovan JL. Analysis of clinical decision-making in multi-disciplinary cancer teams. Ann Oncol. 2006;17:457-460. PMID: 16322114

Taylor C, Munro AJ, Glynne-Jones R, et al. Multidisciplinary team working in cancer: what is the evidence? BMJ. 2010;340:c951. PMID: 20332315

Bazot M, Jarboui L, Ballester M, Touboul C, Thomassin-Naggara I, Daraï E. The value of MRI in assessing parametrial involvement in endometriosis. Hum Reprod. 2012;27:2352-2358. PMID: 22693170

Rousset P, Florin M, Bharwani N, et al. Deep pelvic infiltrating endometriosis: MRI consensus lexicon and compartment-based approach from the ENDOVALIRM group. Diagn Interv Imaging. 2023;104:95-112. PMID: 36404224

Thomassin-Naggara I, Monroc M, Chauveau B, et al. Multicenter external validation of the deep pelvic endometriosis index magnetic resonance imaging score. JAMA Netw Open. 2023;6:e231168. PMID: 37140921

Thomassin-Naggara I, Zoua CS, Bazot M, Monroc M, Roman H, Razakamanantsoa L, Rousset P; ENDOVALIRM study group. Diagnostic MRI for deep pelvic endometriosis: towards a standardized protocol? Eur Radiol. 2024;34:7705-7715. PMID: 38958695

***Ringe***

Kim HA, Kim KA, Choi JI, et al. Comparison of biannual ultrasonography and annual non-contrast liver magnetic resonance imaging as surveillance tools for hepatocellular carcinoma in patients with liver cirrhosis (MAGNUS-HCC): a study protocol. BMC Cancer. 2017;17:877. PMID: 29268722

Pozzi-Mucelli RM, Rinta-Kiikka I, Wünsche K, et al. Pancreatic MRI for the surveillance of cystic neoplasms: comparison of a short with a comprehensive imaging protocol. Eur Radiol. 2017;27:41-50. PMID: 27246720

Tzartzeva K, Obi J, Rich NE, et al. Surveillance imaging and alpha fetoprotein for early detection of hepatocellular carcinoma in patients with cirrhosis: a meta-analysis. Gastroenterology. 2018;154:1706-1718. PMID: 29425931

An C, Kim DY, Choi JY, et al. Noncontrast magnetic resonance imaging versus ultrasonography for hepatocellular carcinoma surveillance (MIRACLE-HCC): study protocol for a prospective randomized trial. BMC Cancer. 2018;18:915. PMID: 30249190

An JY, Peña MA, Cunha GM, Booker MT, Taouli B, Yokoo T, Sirlin CB, Fowler KJ. Abbreviated MRI for hepatocellular carcinoma screening and surveillance. Radiographics. 2020;40:1916-1931. PMID: 33136476

Brunsing RL, Fowler KJ, Yokoo T, Cunha GM, Sirlin CB, Marks RM. Alternative approach of hepatocellular carcinoma surveillance: abbreviated MRI. Hepatoma Res. 2020;6:59. PMID: 33381651

Vietti Violi N, Lewis S, Liao J, et al. Gadoxetate-enhanced abbreviated MRI is highly accurate for hepatocellular carcinoma screening. Eur Radiol. 2020;30:6003-6013. PMID: 32588209

Whang S, Choi MH, Choi JI, Youn SY, Kim DH, Rha SE. Comparison of diagnostic performance of non-contrast MRI and abbreviated MRI using gadoxetic acid in initially diagnosed hepatocellular carcinoma patients: a simulation study of surveillance for hepatocellular carcinomas. Eur Radiol. 2020;30:4150-4163. PMID: 32166493

Kim DH, Choi SH, Shim JH, et al. Meta-analysis of the accuracy of abbreviated magnetic resonance imaging for hepatocellular carcinoma surveillance: non-contrast versus hepatobiliary phase-abbreviated magnetic resonance imaging. Cancers (Basel). 2021;13:2975. PMID: 34198589

Gupta P, Soundararajan R, Patel A, Kumar-M P, Sharma V, Kalra N. Abbreviated MRI for hepatocellular carcinoma screening: A systematic review and meta-analysis. J Hepatol. 2021; 75:108-119. PMID: 33548385

Yoo J, Min JH, Lee DH, Hur BY, Kim SW, Kim E. Abbreviated magnetic resonance imaging with breath-hold three-dimensional magnetic resonance cholangiopancreatography: assessment of malignant risk of pancreatic intraductal papillary mucinous neoplasm. J Magn Reson Imaging. 2021;54:1177-1186. PMID: 3377924

Delaney FT, Cronin CG. Growing evidence for the use of an abbreviated magnetic resonance imaging protocol in the surveillance of pancreatic intraductal papillary mucinous neoplasms. J Magn Reson Imaging. 2021;54:1365-1366. PMID: 34121265

Singal AG, Haaland B, Parikh ND, et al. Comparison of a multitarget blood test to ultrasound and alpha-fetoprotein for hepatocellular carcinoma surveillance: Results of a network meta-analysis. Hepatol Commun. 2022;6:2925-2936. PMID: 35945907

Brandi N, Renzulli M. Towards a simplified and cost-effective diagnostic algorithm for the surveillance of intraductal papillary mucinous neoplasms (IPMNs): can we save contrast for later? Cancers (Basel). 2024;16:905. PMID: 38473267

European Association for the Study of the Liver. EASL clinical practice guidelines on the management of hepatocellular carcinoma. J Hepatol. 2025;82:315-374. PMID: 39690085

***Scheenen***

Tenbergen CJA, Fortuin AS, van Asten JJA, et al. The potential of iron oxide nanoparticle-enhanced MRI at 7 T compared with 3 T for detecting small suspicious lymph nodes in patients with prostate cancer. Invest Radiol. 2024;59:519-525. PMID: 38157433

Pohmann R, Speck O, Scheffler K. Signal-to-noise ratio and MR tissue parameters in human brain imaging at 3, 7, and 9.4 tesla using current receive coil arrays. Magn Reson Med. 2016;75:801-809. PMID: 25820458

Maas MC, Vos EK, Lagemaat MW, et al. Feasibility of T2 -weighted turbo spin echo imaging of the human prostate at 7 tesla. Magn Reson Med. 2014;71:1711-1719. PMID: 23798333

Philips BWJ, Stijns RCH, Rietsch SHG, et al. USPIO-enhanced MRI of pelvic lymph nodes at 7-T: preliminary experience. Eur Radiol. 2019;29:6529-6538. PMID: 31201525

Maatman IT, Schulz J, Ypma S, et al. Free-breathing high-resolution respiratory-gated radial stack-of-stars magnetic resonance imaging of the upper abdomen at 7 T. NMR Biomed. 2024;37:e5180. PMID: 38775032

Van de Moortele PF, Akgun C, Adriany G, et al. B(1) destructive interferences and spatial phase patterns at 7 T with a head transceiver array coil. Magn Reson Med. 2005;54:1503-1518. PMID: 16270333

Metzger GJ, Snyder C, Akgun C, Vaughan T, Ugurbil K, Van de Moortele PF. Local B1+ shimming for prostate imaging with transceiver arrays at 7T based on subject-dependent transmit phase measurements. Magn Reson Med. 2008;59:396-409. PMID: 18228604

Orzada S, Maderwald S, Poser BA, et al. Time-interleaved acquisition of modes: an analysis of SAR and image contrast implications. Magn Reson Med. 2012;67:1033-1041. PMID: 21858867

Maatman IT, Ypma S, Kachelrieß M, et al. Single-spoke binning: Reducing motion artifacts in abdominal radial stack-of-stars imaging. Magn Reson Med. 2023;89:1931-1944. PMID: 36594436

Lagemaat MW, Breukels V, Vos EK, et al. (1)H MR spectroscopic imaging of the prostate at 7T using spectral-spatial pulses. Magn Reson Med. 2016;75:933-945. PMID: 25943445

Philips BWJ, Fortuin AS, Orzada S, et al. High-resolution MR imaging of pelvic lymph nodes at 7 Tesla. Magn Reson Med. 2017;78:1020-1028. PMID: 27714842

***Shampain***

Gutzeit A, Binkert CA, Koh DM, et al. Evaluation of the anti-peristaltic effect of glucagon and hyoscine on the small bowel: comparison of intravenous and intramuscular drug administration. Eur Radiol 2012;22:1186-1194. PMID: 22270141

Czeyda-Pommersheim F, Kalb B, Costello J, et al. MRI in pelvic inflammatory disease: a pictorial review. Abdom Radiol (N Y) 2017;42:935–50. PMID: 27933478

Nougaret S, Nikolovski I, Paroder V, et al. MRI of tumors and tumor mimics in the female pelvis: anatomic pelvic space-based approach. Radiographics 2019;39:1205–1229. PMID: 31283453

Sadowski EA, Rockall AG, Maturen KE, et al. Adnexal lesions: Imaging strategies for ultrasound and MR imaging. Diagn Interv Imaging 2019;100:635-646. PMID: 30177450

Olpin JD, Strachowski L. Imaging of acute pelvic pain: nonpregnant. Radiol Clin North Am 2020;58:329–45. PMID: 30177450

***Sheth***

Motosugi U, Ichikawa T, Morisaka H, et al. Detection of pancreatic carcinoma and liver metastases with gadoxetic acid–enhanced MR imaging: Comparison with contrast-enhanced multi–detector row CT. Radiology. 2011;260:446–453. PMID: 21693662

Chandarana H, Heacock L, Rakheja R, et al. Pulmonary nodules in patients with primary malignancy: comparison of hybrid PET/MR and PET/CT imaging. Radiology. 2013;268:874-881. PMID: 23737537

​

Rauscher I, Eiber M, Fürst S, et al. PET/MR imaging in the detection and characterization of pulmonary lesions: technical and diagnostic evaluation in comparison to PET/CT. J Nucl Med. 2014;55:724-729. PMID: 24652827

Burris NS, Johnson KM, Larson PEZ, et al. Detection of small pulmonary nodules with ultrashort echo time sequences in oncology patients by using a PET/MR system. Radiology. 2015;278:239-246. PMID: 26133050

Vreugdenburg TD, Ma N, Duncan JK, Riitano D, Cameron AL, Maddern GJ. Comparative diagnostic accuracy of hepatocyte-specific gadoxetic acid (Gd-EOB-DTPA) enhanced MR imaging and contrast enhanced CT for the detection of liver metastases: a systematic review and meta-analysis. Int J Colorectal Dis. 2016;31:1739-1749. PMID: 27682648

Raad RA, Friedman KP, Heacock L, Ponzo F, Melsaether A, Chandarana H. Outcome of small lung nodules missed on hybrid PET/MRI in patients with primary malignancy. J Magn Reson Imaging. 2016;43:504-511. PMID: 26192731

Kang B, Lee JM, Song YS, et al. Added value of integrated whole-body PET/MRI for evaluation of colorectal cancer: comparison with contrast-enhanced MDCT. AJR Am J Roentgenol. 2016;206:W10-20. PMID: 26700358

Kim C, Kim SY, Kim M-J, et al. Clinical impact of preoperative liver MRI in the evaluation of synchronous liver metastasis of colon cancer. Eur Radiol. 2018;28:4234-4242. PMID: 29691635

Bailey JJ, Jordan EJ, Burke C, et al. Does extended PET acquisition in PET/MRI rectal cancer staging improve results? AJR Am J Roentgenol. 2018;211:896-900. PMID: 30106609

Wang ZJ, Behr S, Consunji MV, et al. Early response assessment in pancreatic ductal adenocarcinoma through integrated PET/MRI. AJR Am J Roentgenol. 2018;211:1010–1019.​ PMID: 30063366

Chen B-B, Tien Y-W, Chang M-C, et al. Multiparametric PET/MR imaging biomarkers are associated with overall survival in patients with pancreatic cancer. Eur J Nucl Med Mol Imaging. 2018;45:1205–1217.​ PMID: 29476229

Hope TA, Kassam Z, Loening A, McNamara MM, Paspulati R. The use of PET/MRI for imaging rectal cancer. Abdom Radiol. 2019;44:3559-3568. PMID: 31201431

***Shimron***

Pipe JG. Motion correction with PROPELLER MRI: application to head motion and free-breathing cardiac imaging. Magn Reson Med. 1999;42:963-969. PMID: 10542356

Muckley MJ, Riemenschneider B, Radmanesh A, et al. Results of the 2020 fastMRI Challenge for Machine Learning MR Image Reconstruction. IEEE Trans Med Imaging. 2021;40:2306-2317. PMID: 33929957

Kuestner T, Pan J, Qi H, et al. LAPNet: Non-rigid registration derived in k-space for magnetic resonance imaging. IEEE Trans Med Imaging. 2021;40:3686-3697. PMID: 34242163

Wang F, Qi H, Goyeneche A, Heckel R, Lustig M, Shimron E. K-band: Self-supervised MRI reconstruction via stochastic gradient descent over K-space subsets. ArXiv 2024; arXiv:2308.02958.

Ghoul A, Pan J, Lingg A, et al. Attention-aware non-rigid image registration for accelerated MR imaging. IEEE Trans Med Imaging. 2024;43:3013-3026. PMID: 39088484

Shetty AS, Ludwig DR, Ippolito JE, Andrews TJ, Narra VR, Fraum TJ. Low-field-strength body MRI: challenges and opportunities at 0.55 T. Radiographics. 2023;43:e230073. PMID: 37917537

***Yin***

Ratziu V, Charlotte F, Heurtier A, et al. Sampling variability of liver biopsy in nonalcoholic fatty liver disease. Gastroenterology. 2005;128:1898-906. PMID: 15940625

Arani A, Murphy MC, Glaser KJ, et al. Measuring the effects of aging and sex on regional brain stiffness with MR elastography in healthy older adults. Neuroimage. 2015;111:59-64. PMID: 25698157

Chen J, Martin-Mateos R, Li J, et al. Multiparametric magnetic resonance imaging/magnetic resonance elastography assesses progression and regression of steatosis, inflammation, and fibrosis in alcohol-associated liver disease. Alcohol Clin Exp Res. 2021;45:2103-2117. PMID: 34486129

Kostallari E, Wei B, Sicard D, et al. Stiffness is associated with hepatic stellate cell heterogeneity during liver fibrosis. Am J Physiol Gastrointest Liver Physiol. 2022;322:G234-G246. PMID: 34941452

Kennedy P, Stocker D, Carbonell G, et al. MR elastography outperforms shear wave elastography for the diagnosis of clinically significant portal hypertension. Eur Radiol. 2022;32:8339-8349. PMID: 35727321

Younossi ZM, Golabi P, Paik JM, Henry A, Van Dongen C, Henry L. The global epidemiology of nonalcoholic fatty liver disease (NAFLD) and nonalcoholic steatohepatitis (NASH): a systematic review. Hepatology. 2023;77:1335-1347. PMID: 36626630

Hojo E, Sui Y, Shan X, et al. MR elastography-based slip interface imaging (SII) for functional assessment of myofascial interfaces: A feasibility study. Magn Reson Med. 2024;92:676-687. PMID: 38523575

Li J, Obrzut M, Chen J, et al. Free-breathing hepatic 2D magnetic resonance elastography. Magn Reson Med. 2025;93:2434-2443. PMID: 40033890
